# A DNA barcode library for 5,200 German flies and midges (Insecta: Diptera) and its implications for metabarcoding‐based biomonitoring

**DOI:** 10.1111/1755-0998.13022

**Published:** 2019-05-14

**Authors:** Jérôme Morinière, Michael Balke, Dieter Doczkal, Matthias F. Geiger, Laura A. Hardulak, Gerhard Haszprunar, Axel Hausmann, Lars Hendrich, Ledis Regalado, Björn Rulik, Stefan Schmidt, Johann‐Wolfgang Wägele, Paul D. N. Hebert

**Affiliations:** ^1^ SNSB‐Zoologische Staatssammlung München Germany; ^2^ Zoological Research Museum Alexander Koenig ‐ Leibniz Institute for Animal Biodiversity Bonn Germany; ^3^ Centre for Biodiversity Genomics University of Guelph Guelph Ontario Canada

**Keywords:** barcode library, biodiversity monitoring, *CO1*, cryptic diversity, Diptera, DNA barcoding, Germany, metabarcoding, mitochondrial DNA

## Abstract

This study summarizes results of a DNA barcoding campaign on German Diptera, involving analysis of 45,040 specimens. The resultant DNA barcode library includes records for 2,453 named species comprising a total of 5,200 barcode index numbers (BINs), including 2,700 *COI* haplotype clusters without species‐level assignment, so called “dark taxa.” Overall, 88 out of 117 families (75%) recorded from Germany were covered, representing more than 50% of the 9,544 known species of German Diptera. Until now, most of these families, especially the most diverse, have been taxonomically inaccessible. By contrast, within a few years this study provided an intermediate taxonomic system for half of the German Dipteran fauna, which will provide a useful foundation for subsequent detailed, integrative taxonomic studies. Using DNA extracts derived from bulk collections made by Malaise traps, we further demonstrate that species delineation using BINs and operational taxonomic units (OTUs) constitutes an effective method for biodiversity studies using DNA metabarcoding. As the reference libraries continue to grow, and gaps in the species catalogue are filled, BIN lists assembled by metabarcoding will provide greater taxonomic resolution. The present study has three main goals: (a) to provide a DNA barcode library for 5,200 BINs of Diptera; (b) to demonstrate, based on the example of bulk extractions from a Malaise trap experiment, that DNA barcode clusters, labelled with globally unique identifiers (such as OTUs and/or BINs), provide a pragmatic, accurate solution to the “taxonomic impediment”; and (c) to demonstrate that interim names based on BINs and OTUs obtained through metabarcoding provide an effective method for studies on species‐rich groups that are usually neglected in biodiversity research projects because of their unresolved taxonomy.

## 1 INTRODUCTION

Recent evidence for major declines in insect populations has provoked intense public concern. Detailed research on economically important groups, such as pollinators, have linked declines in wild bees to pesticide contamination, climate change, habitat fragmentation and degradation (Potts et al., 2010; Vanbergen & the Insect Pollinators Initiative, 2013). Other studies using mass collecting methods suggest the declines may be general, as evidenced by reductions in the biomass of flying insects by 75% over a few decades (Hallmann et al., 2017; Sorg, Schwan, Stenmans, & Müller, 2013) or even within a few years (Lister & Garcia, 2018). However, the evidence for general declines has failed to ascertain if impacts span all insect groups and all size ranges. The failure to track the status of individual lineages reflects the fact that despite advances in taxonomic practices (e.g., integrative taxonomy), our knowledge of most insect species is limited (Brix, Leese, Riehl, & Kihara, 2015; Cruaud, Rasplus, Rodriguez, & Cruaud, 2017; Pante, Schoelinck, & Puillandre, 2014; Riedel, Sagata, Suhardjono, Tänzler, & Balke, 2013; Wheeler, Raven, & Wilson, 2004). Even in Germany, a country with more than 250 years of taxonomic and faunistic research activity, many groups remain poorly known. This gap, which hampers ecological baseline research, is particularly serious for the two hyperdiverse insect orders, the Diptera and Hymenoptera (Geiger, Moriniere, et al., 2016; Klausnitzer, 2006). With at least 9,500 (Schumann, Bährmann, & Stark, 1999; Schumann, Doczkal, & Ziegler, 2011) and 9,600 (Dathe & Blank, 2004) recorded species in Germany, respectively, these two groups comprise over half of its insect alpha diversity (Völkl, Blick, Kornacker, & Martens, 2004). Moreover, it is likely that the true diversity of these two groups is seriously underestimated, a conclusion reinforced by the extraordinarily high numbers of DNA barcode clusters retrieved by simultaneous analysis of arthropods using high‐throughput sequencing (HTS; metabarcoding) from insect collections at single monitoring sites (Morinière et al., 2016). As only about 1,000 (Santos, Sampronha, & Santos, 2017) new species of Diptera are described each year from the million or more species awaiting description, the taxonomic impediment in this group will not be resolved without the adoption of new approaches, such as modern molecular genetic methods and integrative taxonomy (Fujita, Leache, Burbrink, McGuire, & Moritz, 2012; Padial, Miralles, Riva, & Vences, 2010; Schlick‐Steiner, Arthofer, & Steiner, 2014; Schlick‐Steiner et al., 2010).

The known dipteran fauna of Germany includes roughly half of the almost 20,000 species recorded for Europe (as defined in Fauna Europaea, https://fauna-eu.org/; Pape, 2009). Although this is the highest number of Diptera species recorded from any European country, the inventory is certainly very incomplete. A recent checklist for the Empidoidea of Germany (Meyer & Stark, 2015) added 123 species new to Germany, an increase of 12.5%, Jaschhof (2009) added 34 species of Lestremiinae, an increase of 24.3%, and the collecting efforts for different barcoding campaigns resulted in more than 100 species from various families new to Germany among the identified material (Reimann & Rulik, 2015; Heller & Rulik, 2016; B. Rulik unpublished, D. Doczkal unpublished), with many more expected among the unidentified material. Rapid progress in inventorying is hampered by a lack of experts, also known as the taxonomic impediment (de Carvalho et al., 2007). For example, the German Dipterologist's working group (http://www.ak-diptera.de/index.htm, Accessed 18 December 2018) shows that experts were lacking for one‐third of the dipteran families, and that most other families had just one or two experts, often voluntary (i.e., unpaid) taxonomists (in the sense defined by Fontaine et al., 2012). A few families such as the Culicidae (https://mueckenatlas.com/), the Asilidae (Wolff, Gebel, & Geller‐Grimm, 2018) and the Syrphidae (Ssymank, Doczkal, Rennwald, & Dziock, 2011) are fairly well explored, but several of the species‐richest families (e.g., Cecidomyiidae, Ceratopogonidae, Phoridae, Chloropidae, Sphaeroceridae, Anthomyiidae) have received little attention. Malaise traps are widely used as method of choice to collect arthropods and especially flying insects for biodiversity assessments in terrestrial ecosystems, with Diptera being among the most commonly caught taxa (Doczkal, 2017; Hallmann et al., 2017; Hebert et al., 2016; Karlsson, Pape, Johansson, Liljeblad, & Ronquist, 2005; Matthews & Matthews, 1971; Ssymank et al., 2018). The analysis of specimens from two Malaise traps deployed for a single summer in Germany within the Global Malaise Trap Program (GMTP; http://biodiversitygenomics.net/projects/gmp/) revealed similar trends; here Diptera was the most diverse order being represented by 2,500 species, slightly more than half of all the species that were collected and 70.3% of all individuals (26,189) that were analysed (Geiger, Moriniere, et al., 2016).

Taxonomists working on Diptera have long been well aware of the immense number of undescribed species (Bickel et al., 2009) with estimates of global Diptera species diversity ranging from 400,000 to 800,000 species compared with ~160,000 named species (Borkent et al., 2018; Pape, Blagoderov, & Mostovski, 2011). Hebert et al. (2016), applying DNA barcoding to Canadian insects, proposed that the actual number of species could be much higher, suggesting the possible presence of 1.8 million species in just one family, the Cecidomyiidae (gall midges) alone. Although this estimate may be too high, it is very likely that this single family includes more species than are currently described for the order.

At a time when hundreds or possibly thousands of species become extinct each year (Chivian & Bernstein, 2008), a comprehensive species inventory based on accurately identified specimens represents the foundation for all conservation and biodiversity initiatives. However, the inventory of biodiversity cannot be completed through morphological approaches alone. Both the speed and costs associated with sequence characterization of a standardized DNA fragment can be improved using DNA barcoding. Usually DNA barcoding studies provide a basis for establishing the reference sequence libraries required to identify specimens of known species (Gwiazdowski, Foottit, Maw, & Hebert, 2015; Hebert, Cywinska, Ball, & Dewaard, 2003). Herein we additionally show that it is also an efficient method for registering unknown and taxonomically challenging species—so called “dark taxa” (Page, 2016). Sequenced taxa can subsequently be associated with established binomens by taxonomic specialists using a reverse taxonomy approach, based on accurately identified museum specimens (ideally type specimens) and expert knowledge. During this process, specimens that belong to unnamed molecular character‐based units (operational taxonomic units [OTUs] or barcode index numbers [BINs]) will either be referenced to known species or they may represent overlooked species that are new to science (Geiger, Moriniere, et al., 2016). A curated and comprehensive DNA barcode reference library enables fast and reliable species identifications in those many cases where time, personnel and taxonomic expertise are limited. Furthermore, such a library also supports large‐scale biodiversity monitoring that relies upon metabarcoding bulk samples (Hajibabaei, Shokralla, Zhou, Singer, & Baird, 2011; Hajibabaei, Spall, Shokralla, & Konynenburg, 2012; Shokralla, Spall, Gibson, & Hajibabaei, 2012), like those obtained from Malaise traps (Gibson et al., 2014; Leray & Knowlton, 2015; Morinière et al., 2016; Yu et al., 2012).

The results reported in this study derive from two major DNA barcoding campaigns: “Barcoding Fauna Bavarica” (BFB, http://www.faunabavarica.de; Haszprunar, 2009) and the “German Barcode of Life” project (GBOL, http://www.bolgermany.de; Geiger, Astrin, et al., 2016). Since 2009, DNA barcodes from over 23,000 German species of Metazoa have been assembled, reflecting the analysis of nearly 250,000 specimens that are curated at the SNSB‐Zoologische Staatssammlung München (ZSM, see http://www.barcoding-zsm.de) and ~180,000 specimens curated at the Zoologisches Forschungsmuseum Alexander Koenig Bonn (ZFMK). These records represent a major contribution to the global DNA barcode library that is maintained in the Barcode of Life Data System (BOLD, http://www.boldsystems.org; Ratnasingham & Hebert, 2007). Currently, the DNA barcode library created by the ZSM researchers represents the second‐most comprehensive library of any nation. Previous studies have reported on barcoding results for Coleoptera (Hendrich et al., 2015; Raupach, Hannig, Moriniere, & Hendrich, 2016; Raupach, Hannig, Morinière, & Hendrich, 2018; Rulik et al., 2017), Ephemeroptera, Plecoptera and Trichoptera (Morinière et al., 2017), Heteroptera (Havemann et al., 2018; Raupach et al., 2014), Hymenoptera (Schmid‐Egger et al., 2019; Schmidt, Schmid‐Egger, Morinière, Haszprunar, & Hebert, 2015; Schmidt et al., 2017), Lepidoptera (Hausmann, Haszprunar, & Hebert, 2011; Hausmann, Haszprunar, Segerer, et al., 2011), Neuroptera (Morinière et al., 2014), Orthoptera (Hawlitschek et al., 2017), Araneae and Opiliones (Astrin et al., 2016), and Myriapoda (Spelda, Reip, Oliveira Biener, & Melzer, 2011; Wesener et al., 2015). Concerning DNA barcoding studies performed for Diptera, no comprehensive study encompassing this entire highly diverse order has been published, but data have been used to revise smaller units thereof: for example, for Calliphoridae (Jordaens et al., 2013; Nelson, Wallman, & Dowton, 2007; Reibe, Schmitz, & Madea, 2009), Ceratopogonidae (Stur & Borkent, 2014), Chironomidae (Carew, Pettigrove, Cox, & Hoffmann, 2007; Carew, Pettigrove, & Hoffmann, 2005; Cranston et al., 2013; Ekrem, Stur, & Hebert, 2010; Ekrem, Willassen, & Stur, 2007; Montagna, Mereghetti, Lencioni, & Rossaro, 2016; Pfenninger, Nowak, Kley, Steinke, & Streit, 2007; Sinclair & Gresens, 2008; Stur & Ekrem, 2011), Culicidae (Ashfaq et al., 2014; Cywinska, Hunter, & Hebert, 2006; Kumar, Rajavel, Natarajan, & Jambulingam, 2007; Versteirt et al., 2015; Wang et al., 2012), Hybotidae (Nagy, Sonet, Mortelmans, Vandewynkel, & Grootaert, 2013), Muscidae (Renaud, Savage, & Adamowicz, 2012), Psychodidae (Gutiérrez, Vivero, Vélez, Porter, & Uribe, 2014; Krüger, Strüven, Post, & Faulde, 2011; Kumar, Srinivasan, & Jambulingam, 2012; Nzelu et al., 2015), Sciaridae (Eiseman, Heller, & Rulik, 2016; Heller, Köhler, Menzel, Olsen, & Gammelo, 2016; Heller & Rulik, 2016; Latibari, Moravvej, Heller, Rulik, & Namaghi, 2015; Ševčík, Kaspřák, & Rulik, 2016), Simuliidae (Rivera & Currie, 2009), Syrphidae (Jordaens et al., 2015) and Tachinidae (Pohjoismäki, Kahanpää, & Mutanen, 2016).

This publication presents the first results of the Diptera campaign and it provides coverage for 5,200 BINs (Ratnasingham & Hebert, 2013). It covers ~55% of the known Diptera fauna from Germany. According to the checklist of German Diptera (Schumann et al., 1999) and the three additions published so far (Schumann, 2002, 2004, 2010) 9,544 species of Diptera have been recorded from Germany. The Diptera library now includes a total of 2,453 reliable species identifications, and 2,700 BINs, which possess either interim species names or just higher‐level taxonomy (genus or family; “dark taxa”). Although it has been shown that BINs correspond closely to biological species of most insect orders (Hausmann et al., 2013), there are other studies reporting difficulties in determining species through DNA barcodes within Diptera. In particular, well‐studied groups such as the syrphids represent a problem, because here additional genes for a clear type assignment must be consulted in many genera (Mengual, Ståhls, Vujić, & Marcos‐Garcia, 2006; Rojo, Ståhls, Pérez‐Bañón, & Marcos‐García, 2006). Further examples of problems in species delineation due to barcode gaps, at least for some genera, are the Tachinidae and the Calliphoridae (Nelson et al., 2012; Pohjoismäki et al., 2016; Whitworth, Dawson, Magalon, & Baudry, 2007). In one of the few studies dealing with DNA barcoding in Diptera it was shown, that less than 70% of a composition of about 450 species covering 12 families of Diptera could be reliably identified by DNA barcoding, as there was wide overlap between intra‐ and interspecific genetic variability on the *COI* gene (Meier, Shiyang, Vaidya, & Ng, 2006). However we find that more than 88% of the studied species, identified based on morphology or BIN matches to the BOLD database, can be unambiguously identified using their DNA barcode sequences. BINs enable the creation of an interim taxonomic system in a structured, transparent and sustainable way and thus become a valuable foundation for subsequent detailed, integrative taxonomic studies. Furthermore, the BIN system enables analyses that are equivalent to studies based on named species, that is where the underlying specimens are identified by specialists using traditional methods (i.e., morphology). The latter will play a special role in the processing, classification and genetic inventorying of less‐explored “dark taxa,” which have been treated and processed with less priority by previous DNA barcoding activities. Moreover, this automated approach of delineating species is less affected by operational taxonomic biases, so it can provide more objective identifications than conventional approaches (Mutanen et al., 2016; Packer, Gibbs, Sheffield, & Hanner, 2009; Schmidt et al., 2015). Using DNA extracts derived from bulk collections made by Malaise traps, we further demonstrate that species delineation using interim names based on BINs and OTUs constitutes an effective method for biodiversity studies using DNA metabarcoding. As the reference libraries continue to grow and gaps in the species catalogue are subsequently filled, BIN lists assembled by metabarcoding will provide improved taxonomic resolution.

The present study has three main goals: (a) to provide a DNA barcode library for 5,200 BINs of Diptera; (b) to demonstrate, based on the example of bulk extractions from a Malaise trap experiment, that DNA barcode clusters, labelled with globally unique identifiers (such as OTUs and/or BINs), provide a pragmatic, accurate solution to the “taxonomic impediment”; and (c) to demonstrate that interim names based on BINs and OTUs obtained through metabarcoding is an effective method for studies on species‐rich groups that are usually neglected in biodiversity research projects because of their unresolved taxonomy.

## 2 MATERIALS AND METHODS

### 2.1 Fieldwork, specimens and taxonomy

A network of 130 (professional and voluntary) taxonomists and citizen scientists collected and contributed specimens to the DNA barcoding projects, primarily from various German states, but also from surrounding European countries (Austria, Belgium, Czech Republic, France, Italy). Most specimens (94.5%, 42,587 of 45,040 with *COI* sequences >500 bp) were collected by Malaise traps, which were deployed from 2009 to 2016. The study sites included more than 683 localities in state forests, public lands and protected areas such as the Nationalparks “Bayerischer Wald” and “Berchtesgadener Land,” the EU habitats directive site “Landskrone,” as well as alpine regions at altitudes up to 2,926 m (Zugspitze). Detailed information on collection sites and dates is available in Appendix S1. Since 2009, more than five million specimens of Diptera were collected by hand collecting, sweep netting, and by Malaise‐, window‐ and pitfall‐trapping. However, most voucher specimens have been extracted from Malaise trap samples. Twenty to 100 Malaise traps were deployed in each of seven years (2011–2017) mostly across habitats in Bavaria and Baden‐Wurttemberg; one trap was placed in Rhineland‐Palatinate. Samples were screened morphologically to maximize the diversity of species submitted for sequence characterization. Most vouchers were derived from Germany (44,511), but others were collected in France (222), Czech Republic (147), Belgium (106), Austria (70) and other Central European countries (18). All samples and specimens are now stored in the SNSB‐ZSM or ZFMK except for a few held in private collections. From the entire collection, ~3,000,000 specimens of potential interest, most of which derived from the huge Malaise trap experiments in the framework of the GMTP, were identified to family level mostly by D.D. and to a minor extent by B.R. and experienced specialists using appropriate literature (Oosterbroek, 2006 and references therein; Papp & Darvas, 1997, 1998, 2000a, 2000b, Schumann et al., 2011). From this material, 59,000 specimens were submitted for sequence analysis through the DNA barcoding pipeline (including sample preparation, high‐quality imaging and metadata acquisition for each specimen) established at the ZSM to support its involvement in national and international DNA barcoding projects. Most samples (>99%) were stored in 96% EtOH before DNA extraction. Specimen ages generally ranged from 1 to 5 years (43,112 specimens, 96%); only 4% were more than 5 years old. The number of specimens analysed per species ranged from one to 1,356 (i.e., *Megaselia rufa*) (Wood, 1908; see Appendix S1). When taxonomic expertise was available, specimens were sent to specialists to obtain as many species‐level identifications as possible.

### 2.2 Laboratory protocols

A tissue sample was removed from each specimen and transferred into 96‐well plates at the SNSB‐ZSM for subsequent DNA extraction. For specimens with a body length >2 mm a single leg or a leg segment was removed for DNA extraction. The whole voucher was used for some very small specimens (e.g., ≤1 mm, such as small Cecidomyiidae, Chironomidae and Sciaridae), but replacement vouchers from the same locality were retained. In other cases (vouchers from Malaise traps), DNA was extracted from the whole voucher at the CCDB (Guelph, Canada) using “voucher‐recovery” protocols (DeWaard et al., 2019) and the specimens were repatriated to the SNSB‐ZSM and ZFMK for identification and curation. DNA extraction plates with the tissue samples were sent to the Canadian Center for DNA Barcoding (CCDB) where they were processed using standard protocols. All protocols for DNA extraction, PCR amplifications and Sanger sequencing procedures are available online (ccdb.ca/resources/). All samples were PCR‐amplified with a cocktail of standard and modified Folmer primers CLepFolF (5′‐ATTCAACCAATCATAAAGATATTGG) and CLepFolR (5′TAAACTTCTGGATGTCCAAAAAATCA) for the barcode fragment (5′ *COI*; see Hernández‐Triana et al., 2014), and the same primers were employed for subsequent bidirectional Sanger sequencing reactions (see also Ivanova, Dewaard, & Hebert, 2006; deWaard, Ivanova, Hajibabaei, & Hebert, 2008, DeWaard et al., 2019). Voucher information such as locality data, habitat, altitude, collector, identifier, taxonomic classifications, habitus images, DNA barcode sequences, primer pairs and trace files for 40,753 specimens are publicly accessible in the “DS‐DIPBFGBL—A DNA Barcode reference library of German Diptera (BFB—Barcoding Fauna Bavarica & GBOL—German Barcode of Life” data set on BOLD (http://www.boldsystems.org – data set DOI: http://dx.doi.org/10.5883/DS-DIPBFGBL), whereas 4,420 specimen records will be stored in the private data set “DS‐DIPBFGBP—A DNA Barcode reference library of German Diptera (BFB—Barcoding Fauna Bavarica & GBOL—German Barcode of Life)—private records for future publication” for subsequent publication by the authors and associated taxonomists.

### 2.3 Data analysis

Sequence divergences for the *COI*‐5P barcode region (mean and maximum intraspecific variation and minimum genetic distance to the nearest‐neighbouring species) were calculated using the “*Barcode Gap Analysis*” tool on BOLD, employing the Kimura 2‐parameter (K2P) distance metric (Puillandre, Lambert, Brouillet, & Achaz, 2012). The program muscle was applied for sequence alignment restricting analysis to sequences with a minimum length of 500 bp. Neighbour‐joining (NJ) trees were calculated following alignment based on K2P distances. The “BIN Discordance” analysis on BOLD was used to reveal cases where species assigned to different species shared a BIN, and those cases where a particular species was assigned to two or more BINs. Sequences are grouped into clusters of closely similar *COI* barcode sequences, which are assigned a globally unique identifier, termed a “barcode index number” or BIN (Ratnasingham & Hebert, 2013). This system enables tentative species identifications when taxonomic information is lacking. The BIN system involves a three‐step online pipeline, which clusters similar barcode sequences algorithmically into OTUs being “named” by a number. For the majority of studied insect orders, specimens sharing a BIN very often represent a close species‐proxy as delineated by traditional taxonomy (e.g., for Lepidoptera, Hausmann et al., 2013). However, some genera or families throughout the insects exhibit problems with species delineation based on DNA barcodes, due to high intra‐ or low interspecific genetic distances (e.g., cryptic diversity, BIN sharing or the barcode gap; see Hubert & Hanner, 2015). Within the Diptera, this phenomenon has been well documented (Meier et al., 2006), at least in some families, such as calliphorid, syrphid and tachinid species (Mengual et al., 2006; Nelson et al., 2012; Pohjoismäki et al., 2016; Rojo et al., 2006; Whitworth et al., 2007), but may also occur in families of “dark taxa” as well.

Every other “disagreement/conflict” case is the starting point for re‐evaluation of both molecular and morphological data. We follow the concept of Integrative Taxonomy (Fujita et al., 2012; Padial et al., 2010; Schlick‐Steiner et al., 2014, 2010) to infer whether there are previously overlooked species (“cryptic taxa”) in the sample, or whether barcode divergence between species is too low or absent to allow valid species to be delineated using only *COI* characteristics.

### 2.4 Reverse‐taxonomy approach

When sequenced specimens could only be assigned to a category above the species level (family, subfamily or genus), we used interim species names (such as TachIntGen1 sp.BOLD:AAG2112) based on the corresponding BIN, so these specimens could be included in the “Barcode Gap Analysis” in order to provide more comprehensive estimates of the distribution of genetic divergences among both species assigned to Linnaean species and those with BIN assignments. This analysis was conducted on all specimens at the same time after updating the interim taxonomy where necessary. For specimen records, which lack lower taxonomy (e.g., those uploaded only as “Diptera”), we applied the highest “conflict‐free” taxonomy—for example the genus name, when other specimens within that BIN had the same identification—using a BIN match with the public data on BOLD (e.g., *Melanagromyza* sp. BOLD:ACP6151). All specimens, which could not be identified to species or genus level, and where the vouchers were in acceptable condition (e.g., unbroken antennae and/or legs after retrieval from Malaise trap), were selected using the corresponding BINs for identification by taxonomic specialists. Interim names were subsequently moved into the “Voucher status” field in the BOLD metadata tables after all analyses were performed.

### 2.5 Metabarcoding and bioinformatic data analysis

The potential utility of the DNA barcode library for biomonitoring Diptera was tested with field samples, focusing on an early warning system for pest and invasive species based on metabarcoding (L. A. Hardulak et al. in prep). In this study, nine Malaise traps were deployed in the Bayerischer Wald National Park and its surroundings during the vegetated period (May–September) in 2016. Trap bottles were changed twice monthly, producing a total of 90 bulk samples of macroinvertebrates. All specimens were dried and ground with a stainless steel pestle (no size‐sorting step), and tissue lysis of insect powder per trap sample was performed overnight, using a solution of 90% insect lysis buffer and 10% proteinase K. DNA extraction was performed with the DNEasy Blood & Tissue kit (Qiagen). A minibarcode region was amplified by PCR, using forward and reverse NGS primers (Leray et al., 2013) targeting a 313‐bp‐long coding region of mitochondrial *COI*. High‐throughput sequencing was performed on an Illumina MiSeq using version 2 (2 × 250 bp, 500 cycles, maximum of 20 million reads) chemistry at the Sequencing Service Unit of the Ludwig‐Maximilians University (LMU, Munich, Germany; see Appendix S5 for a more detailed metabarcoding protocol).

Sequence processing was performed with the vsearch version 2.4.3 suite (Rognes, Flouri, Nichols, Quince, & Mahé, 2016) and cutadapt version 1.14 (Martin, 2011). Forward and reverse reads in each sample were merged with the vsearch program “fastq_mergepairs” with a minimum overlap of 40 bp, yielding ~313‐bp sequences. Forward and reverse primers were removed with cutadapt, using the “discard_untrimmed” option to discard sequences for which primers were not detected at ≥90% identity. Quality filtering was done with the “fastq_filter” in vsearch, keeping sequences with zero expected errors (“fastq_maxee” 1). Sequences were dereplicated with “derep_fulllength,” first at the sample level, and then concatenated into a fasta file, which was then dereplicated. Chimeric sequences were removed from the fasta file using “uchime_denovo.” The remaining sequences were then clustered into OTUs at 97% identity employing “cluster_size,” a greedy, centroid‐based clustering program. OTUs were blasted against the Diptera database downloaded from BOLD including taxonomy and BIN information in geneious (version 9.1.7; Biomatters) following the methods described in Morinière et al. (2016). The resulting csv file, which included BIN, Hit‐%‐ID value, family, genus and species information for each out, was exported from Geneious and combined with the OTU table generated by the bioinformatic pipeline. The combined results table was then filtered by Hit‐%‐ID value and total read numbers per OTU. All entries with identifications below 97% and total read numbers below 0.01% of the summed reads per sample were removed from the analysis. OTUs were then assigned to the respective BIN (Appendix S2). Presence–absence overviews of selected Diptera taxa (BINs) within the metabarcoding study were created; one‐sided Pearson correlation coefficients were calculated to estimate the percentage of “dark taxa” with mid‐range body size versus the number of species reported in Germany, both with the inclusion and with the exclusion of families with 0% “dark taxa.” (r version 3.4.4 [2018–03‐15], R Core Team, 2018).

## 3 RESULTS

### 3.1 DNA barcoding/developing a reference library

**Figure 1:**
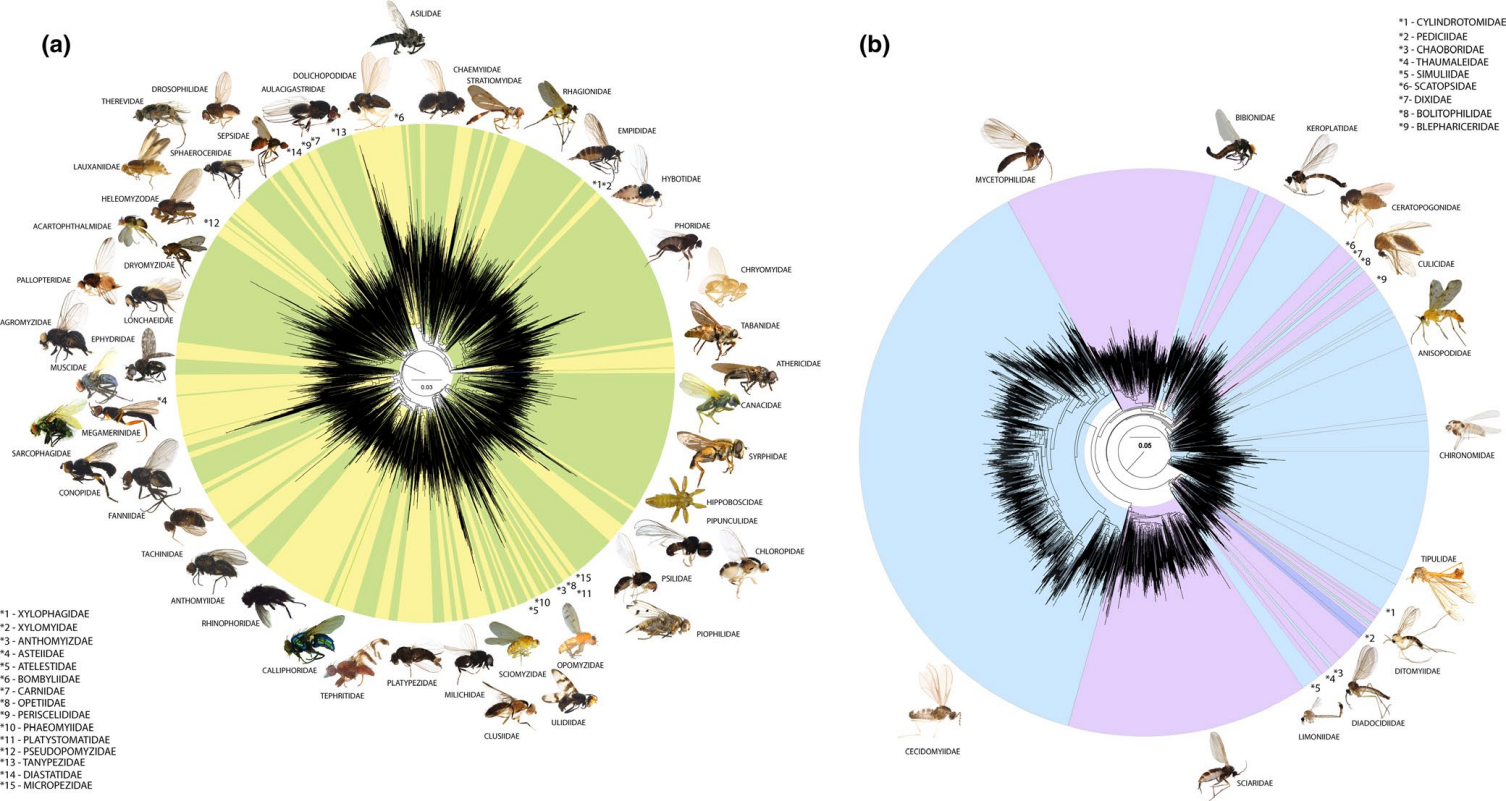
Illustrative circular neighbour‐joining (NJ) trees for (a) all Brachycera and (b) all Nemtatocera within the Diptera barcode library; each line in the trees corresponds to one barcode index number (BIN). NJ tree calculations were performed on the BOLD database. A more detailed observation of the BIN diversity for each family can be studied within the Krona graph within the supporting information (Figure S2) [Colour figure can be viewed at http://www.wileyonlinelibrary.com]

From the 59,102 specimens submitted for Sanger sequencing, 50,963 *COI*‐5P sequences (86.23%) were recovered. Length of the recovered sequence varied with the sequencing protocol; 12.54% (7,410 specimens) were bidirectionally sequenced and yielded a full‐length (658 bp) barcode while the rest (43,533) were unidirectionally sequenced yielding 69.95% (41,339) with sequences <658 to >500 bp and 3.75% (2,214 specimens) with sequences <500 bp. No sequence information was recovered from 13.77% (8,139) of the specimens. Barcode recovery was most successful for EtOH‐preserved specimens less than 10 years old. For the subsequent analyses we selected 45,040 specimens with high‐quality DNA barcode sequences (≥500 bp), which fulfilled the requirements for being assigned to a BIN. This data set included ~5,200 BINs (2,500 were assigned a total of 2,453 Linnean species while 2,700 lacked a species designation, 52.4% of the data set). These BINs included one or more representatives from 88 of the 117 (75%) dipteran families known from Germany (Figure 1, Table 1; Appendix S3, Krona graph in Figure S2). More than one‐third (1,829) of the BINs were new to BOLD.

**Table 1:** Families of Diptera reported in Germany. Information on BIN count, and on the numbers of named and unnamed species within the reference database

Infraorder	Family	Species reported in Germany	BINs	Ratio barcoded/species (%)	Size (mm)	Total number of taxa/with barcode	Unnamed/with barcode	% of dark taxa
Brachycera	Acartophthalmidae	2	1	50	1.0–2.5	2	0	0
Brachycera	Acroceridae	11	0	0	2.5–20.0	N/A	N/A	N/A
Brachycera	Agromyzidae	552	218	39	1.0–6.0	214	149	70
Nematocera	Anisopodidae (& Mycetobiidae)	8	7	88	4.0–12.0	7	2	29
Brachycera	Anthomyiidae	227	188	83	4.0–12.0	178	64	36
Brachycera	Anthomyzidae	14	5	36	1.3–4.5	5	0	0
Brachycera	Asilidae	81	18	22	8.0–20.0	18	6	33
Brachycera	Asteiidae	7	3	43	1.0–3.0	3	0	0
Brachycera	Atelestidae	3	3	100	1.5–3.5	3	0	0
Brachycera	Athericidae	5	3	60	7.5–10.0	3	1	33
Brachycera	Aulacigastridae	1	0	0	2.0–5.0	0	0	N/A
Nematocera	Bibionidae (& Pleciidae)	21	12	57	2.0–15.0	10	2	20
Nematocera	Blephariceridae	7	2	29	3.0–15.0	2	1	50
Nematocera	Bolitophilidae	22	14	64	4.0–7.0	13	7	54
Brachycera	Bombyliidae	40	6	15	1.0–20.0	6	1	17
Brachycera	Braulidae	1	0	0	1.2–2.5	2	0	0
Brachycera	Calliphoridae	62	35	56	4.0–16.0	39	6	15
Brachycera	Camillidae	4	0	0	2.0–3.5	N/A	N/A	N/A
Brachycera	Campichoetidae	3	0	0	2.5–4.0	N/A	N/A	N/A
Brachycera	Canacidae	2	9	450	1.6–5.0	9	1	11
Nematocera	Canthyloscelidae	1	0	0	2.5–9.0	N/A	N/A	N/A
Brachycera	Carnidae	11	7	64	1.0–2.5	7	7	100
Nematocera	Cecidomyiidae	836	927	111	0.5–3.0	926	882	95
Nematocera	Ceratopogonidae	332	131	39	1.0–5.0	128	97	76
Brachycera	Chamaemyiidae	29	17	59	1.0–5.0	17	13	76
Nematocera	Chaoboridae	7	2	29	2.0–10.0	2	0	0
Nematocera	Chironomidae	696	455	65	1.0–10.0	438	286	65
Brachycera	Chloropidae	198	101	51	1.0–5.0	101	59	58
Brachycera	Chyromyidae	5	2	40	0.5–8.0	2	0	0
Brachycera	Clusiidae	9	6	67	1.5–8.0	7	3	43
Brachycera	Coelopidae	2	0	0	2.5–9.0	N/A	N/A	N/A
Brachycera	Coenomyiidae	1	0	0	14.0–20.0	N/A	N/A	N/A
Brachycera	Conopidae	52	9	17	5.0–15.0	9	0	0
Brachycera	Cremifaniidae	1	0	0	1.5–2.6	N/A	N/A	N/A
Brachycera	Cryptochetidae	1	0	0	2.0–4.0	N/A	N/A	N/A
Nematocera	Culicidae	46	8	17	3.0–9.0	7	0	0
Nematocera	Cylindrotomidae	4	1	25	11.0–16.0	1	0	0
Nematocera	Diadocidiidae	4	3	75	3–4.5.0	3	0	0
Brachycera	Diastatidae	6	8	133	2.5–4.0	8	2	25
Nematocera	Ditomyiidae	4	1	25	6.0–8.0	1	0	0
Nematocera	Dixidae	16	4	25	3.0–5.5	4	1	25
Brachycera	Dolichopodidae	356	112	31	1.0–9.0	112	58	52
Brachycera	Drosophilidae	59	28	47	1.5–7.0	28	5	18
Brachycera	Dryomyzidae	3	2	67	5.0–18.0	2	1	50
Brachycera	Eginiidae	1	0	0	2.0–18.0	N/A	N/A	N/A
Brachycera	Empididae (& Brachystomatidae)	383	161	42	1.0–12.0	161	107	66
Brachycera	Ephydridae	177	130	73	1.0–11.0	132	16	12
Brachycera	Fanniidae	56	46	82	2.0–5.0	44	13	30
Brachycera	Gasterophilidae	4	0	0	9.0–16.0	N/A	N/A	N/A
Brachycera	Helcomyzidae	3	0	0	6.0–11.0	N/A	N/A	N/A
Brachycera	Heleomyzidae (& Heteromyzidae)	74	58	78	1.2–12.0	55	26	47
Nematocera	Hesperinidae	1	0	0	4.0–6.0	N/A	N/A	N/A
Brachycera	Hilarimorphidae	2	0	0	2.0–7.0	N/A	N/A	N/A
Brachycera	Hippoboscidae	12	7	58	2.5–10.0	7	1	14
Brachycera	Hybotidae	229	140	61	1.0–6.0	139	83	60
Brachycera	Hypodermatidae	5	0	0	10.0–22	N/A	N/A	N/A
Nematocera	Keroplatidae	60	30	50	4.0–15.0	30	12	40
Brachycera	Lauxaniidae	67	25	37	2.0–7.0	25	11	44
Nematocera	Limoniidae	280	96	34	2.0–11.0	91	50	55
Brachycera	Lonchaeidae	47	16	34	3.0–6.0	16	9	56
Brachycera	Lonchopteridae	9	5	56	2.0–5.0	6	0	0
Brachycera	Megamerinidae	1	1	100	6.0–9.0	1	0	0
Brachycera	Micropezidae	13	5	38	3.0–16.0	4	1	25
Brachycera	Microphoridae	6	0	0	1.5–3.0	N/A	N/A	N/A
Brachycera	Milichiidae	13	17	131	1.0–6.0	16	9	56
Brachycera	Muscidae	317	174	55	2.0–18.0	167	66	40
Nematocera	Mycetophilidae	573	306	53	2.0–15.0	301	89	30
Brachycera	Neottiophilidae	1	0	0	1.5–7.0	N/A	N/A	N/A
Brachycera	Nycteribiidae	8	0	0	1.5–5.0	N/A	N/A	N/A
Brachycera	Odiniidae	9	0	0	2.0–5.0	N/A	N/A	N/A
Brachycera	Oestridae	6	0	0	9.0–18.0	N/A	N/A	N/A
Brachycera	Opetiidae	1	1	100	2.0–5.0	1	0	0
Brachycera	Opomyzidae	15	4	27	2.0–5.0	4	1	25
Brachycera	Otitidae	26	0	0	2.5–11.0	N/A	N/A	N/A
Brachycera	Pallopteridae	16	8	50	2.5–7.0	7	0	0
Nematocera	Pediciidae	36	13	36	5.0–35.0	13	3	23
Brachycera	Periscelididae	6	1	17	1.0–5.0	1	0	0
Brachycera	Phaeomyiidae	3	2	67	3.0–11.0	2	0	0
Brachycera	Phoridae	364	289	79	0.5–6.0	276	166	60
Brachycera	Piophilidae	12	12	100	1.5–7.0	12	4	33
Brachycera	Pipunculidae	111	42	38	2.0–12.0	40	7	18
Brachycera	Platypezidae	23	4	17	1.5–6.0	4	0	0
Brachycera	Platystomatidae	3	2	67	3.0–11.0	2	0	0
Brachycera	Pseudopomyzidae	1	1	100	1.7–2.5	1	0	0
Brachycera	Psilidae	30	12	40	2.5–10.0	12	8	67
Nematocera	Psychodidae	143	51	36	2.0–6.0	50	25	50
Nematocera	Ptychopteridae	8	0	0	7.0–15.0	N/A	N/A	N/A
Brachycera	Pyrgotidae	1	0	0	8.0–9.0	N/A	N/A	N/A
Brachycera	Rhagionidae	35	20	57	2.0–20.0	20	10	50
Brachycera	Rhinophoridae	10	9	90	2.0–11.0	7	1	14
Brachycera	Sarcophagidae	130	49	38	3.0–22.0	49	17	35
Brachycera	Scatophagidae	57	0	0	3.0–12.0	0	0	N/A
Nematocera	Scatopsidae	47	30	64	0.5–4.0	30	24	80
Brachycera	Scenopinidae	3	0	0	2.0–7.0	N/A	N/A	N/A
Nematocera	Sciaridae	342	310	91	1.0–6.0	284	81	29
Brachycera	Sciomyzidae	78	19	24	2.0–14.0	18	4	22
Brachycera	Sepsidae	31	15	48	2.0–6.0	13	1	8
Nematocera	Simuliidae	50	19	38	1.2–6.0	18	9	50
Brachycera	Sphaeroceridae	137	79	58	0.7–5.5	77	31	40
Brachycera	Stratiomyidae	66	21	32	2.0–25.0	22	6	27
Brachycera	Strongylophthalmyiidae	1	0	0	3.0–5.5	N/A	N/A	N/A
Brachycera	Syrphidae	440	242	55	3.5–35.0	297	24	8
Brachycera	Tabanidae	58	46	79	6.0–30.0	45	3	7
Brachycera	Tachinidae	494	214	43	2.0–20.0	211	76	36
Brachycera	Tanypezidae	1	1	100	5.0–8.0	1	0	0
Brachycera	Tephritidae	110	28	25	2.5–10.0	27	5	19
Brachycera	Tethinidae	10	0	0	1.5–3.5	N/A	N/A	N/A
Nematocera	Thaumaleidae	15	13	87	3.0–5.0	13	1	8
Brachycera	Therevidae	32	4	13	2.5–15.0	4	1	25
Brachycera	Thyreophoridae	2	0	0	1.5–7.0	N/A	N/A	N/A
Nematocera	Tipulidae	123	46	37	7.0–35.0	46	15	33
Nematocera	Trichoceridae	18	24	133	3.0–9.0	24	17	71
Brachycera	Trixoscelididae	4	0	0	2.0–4.0	N/A	N/A	N/A
Brachycera	Ulidiidae	4	9	225	2.5–11.0	9	4	44
Brachycera	Xylomyidae	3	1	33	6.0–20.0	1	0	0
Brachycera	Xylophagidae	4	1	25	5.0–11.0	1	0	0

Note

Additional information on the average body size of the specimens in each family is included.

Inspection of the *COI* sequence clusters using NJ trees (created with analytical tools on BOLD) and using the TaxCl‐approach for detecting taxonomic incongruences (Rulik et al., 2017) revealed high congruence with morphology‐based identifications. Among the 2,453 taxa assigned a Linnean binomen based on morphological identifications and “conflict‐free” BIN matches, 88.67% (2,138) were unambiguously discriminated by their *COI* sequences. Another 122 species (4.97%), representing 8.7% of all studied specimens (3,951 individuals), were assigned to more than one BIN, resulting in a total of 255 BINs (Table 1; Appendix S3). For purposes of re‐identification, the species in this subset can also be unambiguously assigned to a current species. For 34 of these taxa, the maximum intraspecific variation (maxISP) was <3% (range: 1.1%–3.0%), cases which may reflect either young sibling species or high intraspecific variation arising from secondary contact between phylogeographical lineages. Another 88 species showed considerably higher divergences with maxISP ranging from 3% to 6% in 48 species and from 6% to 12% in another 40 species, cases that are strong candidates for overlooked cryptic diversity. Most of these cases involved species whose members were assigned to two BINs (112 species), but specimens of nine species were assigned to three BINs and those of one other to four BINs. Another 156 species (6.56%), representing 2.9% of all specimens (1,316 specimens), involved two or more named species that shared a BIN (Table 2). Ten of these species pairs possessed shallow but consistent divergences within the BIN, meaning that *COI* sequences enabled species identification (e.g., *Chrysotoxum bicinctum* Linnaeus, 1758 and *Chrysotoxum festivum* Linnaeus, 1758; *Sericomyia lappona* Linnaeus, 1758 and *Sericomyia silentis* Harris 1776; *Paragus majoranae* Rondani, 1857 and *Paragus pecchiolii*, Rondani, 1857). Interestingly, almost two‐thirds (105/156) of the species exhibiting BIN sharing (168) were hoverflies (Syrphidae), a family that has seen intensive taxonomic study.

**Table 2:** All cases of high intraspecific sequence variation at *COI*; cases of multiple BINs and/or cryptic diversity candidates (CDC)

Family	Species	CDC rank	Mean intraspecific variation	Max. intraspecific variation	BIN
Agromyzidae	*Napomyza cichorii*	*CDC (2)*	2.47	3.71	BOLD:AAP2990
					BOLD:AAX3741
	*Phytomyza continua*	*CDC (2)*	2.84	5.44	BOLD:AAM6330
					BOLD:AAY2701
	*Phytomyza ranunculi*	*CDC (2)*	3.26	6.43	BOLD:AAY3895
					BOLD:ACL2003
Anthomyiidae	*Anthomyia liturata*	*CDC (2)*	0.87	1.98	BOLD:ACE4539
					BOLD:ACE4540
	*Delia nuda*	*CDC (2)*	1.06	1.87	BOLD:ACJ0544
					BOLD:ACJ0545
	*Hydrophoria lancifer*	*CDC (2)*	0.61	3.04	BOLD:AAG2460
					BOLD:ADC1814
	*Pegomya flavifrons*	*CDC (2)*	2.5	8.83	BOLD:AAG2479
					BOLD:AAG6754
	*Pegomya solennis*	*CDC (2)*	0.85	2.67	BOLD:ACD8686
					BOLD:ACM6225
	*Pegomya winthemi*	*CDC (2)*	0.54	5.53	BOLD:AAG1783
					BOLD:ABA6845
Bibionidae	*Bibio clavipes*	*CDC (2)*	1.2	2.46	BOLD:ACC6151
					BOLD:ACR0881
	*Bibio nigriventris*	*CDC (2)*	1	3.13	BOLD:ABX1732
					BOLD:ACU5368
Bolitophilidae	*Bolitophila austriaca*	*CDC (2)*	1.27	2.18	BOLD:AAG4863
					BOLD:ACI5612
Ceratopogonidae	*Brachypogon sociabilis*	*CDC (2)*	1.24	2.31	BOLD:ABW3958
					BOLD:ACE8195
	*Ceratopogon grandiforceps*	*CDC (2)*	2.63	3.94	BOLD:ABW3984
					BOLD:ACP4327
	*Forcipomyia* sp. 4ES	*CDC (2)*	2.18	5.98	BOLD:AAM6200
					BOLD:ACQ8860
Chironomidae	*Brillia bifida*	*CDC (2)*	2.31	6.93	BOLD:AAD7726
					BOLD:ADI4999
	*Cricotopus bicinctus*	*CDC (2)*	1.86	3.2	BOLD:AAI6018
					BOLD:AAT9677
	*Gymnometriocnemus brumalis*	*CDC (2)*	0.5	2.41	BOLD:ACD4501
					BOLD:ACU9207
	*Limnophyes natalensis*	*CDC (2)*	1.51	2.89	BOLD:AAB7361
					BOLD:ACT1270
	*Limnophyes* sp. 4SW	*CDC (2)*	1.49	4.03	BOLD:ACR9428
					BOLD:ACU4225
	*Mesosmittia flexuella*	*CDC (2)*	0.79	2.02	BOLD:ADE7569
					BOLD:ACU4856
	*Orthocladius fuscimanus*	*CDC (2)*	2	2.66	BOLD:AAV5075
					BOLD:ACX3046
	*Parametriocnemus stylatus*	*CDC (2)*	0.76	2.03	BOLD:AAI2687
					BOLD:ACT9205
	*Paraphaenocladius exagitans*	*CDC (3)*	2.54	5.88	BOLD:AAE3719
					BOLD:ACQ4724
					BOLD:ACT8523
	*Paraphaenocladius impensus*	*CDC (4)*	6.85	11.99	BOLD:AAC4200
					BOLD:ACT2714
					BOLD:ACT5784
					BOLD:ACU4175
	*Paratanytarsus laccophilus*	*CDC (2)*	2.09	3.14	BOLD:AAC8842
					BOLD:ACF2457
	*Polypedilum convictum*	*CDC (2)*	2.45	4.61	BOLD:AAW4661
					BOLD:ACT9278
	*Smittia reissi*	*CDC (2)*	1.72	3.47	BOLD:ACS9748
					BOLD:ACU4112
Conopidae	*Myopa testacea*	*CDC (2)*	3.72	3.72	BOLD:AAK8836
					BOLD:AAK8838
Dolichopodidae	*Microphor anomalus*	*CDC (2)*	5.47	11	BOLD:ACH9042
					BOLD:ACH9043
	*Microphor holosericeus*	*CDC (2)*	4.06	12.7	BOLD:ACB6469
					BOLD:ACH6989
Empididae	*Hemerodromia adulatoria*	*CDC (2)*	8.52	8.52	BOLD:ACJ6728
					BOLD:ACJ6729
	*Kowarzia barbatula*	*CDC (2)*	7.21	10.71	BOLD:ACJ6935
					BOLD:ACJ7236
	*Kowarzia tenella*	*CDC (2)*	5.39	10.8	BOLD:ACJ6935
					BOLD:ACJ7236
Ephydridae	*Allotrichoma laterale*	*CDC (2)*	6.44	6.44	BOLD:ABA8753
					BOLD:ACF1575
	*Ditrichophora fuscella*	*CDC (2)*	3.81	7.62	BOLD:ABA8605
					BOLD:ABA8606
	*Ditrichophora palliditarsis*	*CDC (2)*	3.87	6.57	BOLD:AAX8675
					BOLD:ABA8748
	*Halmopota salinarius*	*CDC (2)*	2.43	3.81	BOLD:ABA7826
					BOLD:ABA7827
	*Hydrellia flaviceps*	*CDC (2)*	4.22	6.33	BOLD:ABA8652
					BOLD:ABV8173
	*Philygria flavipes*	*CDC (2)*	1.19	2.03	BOLD:ABA8663
					BOLD:ACK3229
	*Polytrichophora duplosetosa*	*CDC (2)*	2.05	4.11	BOLD:ABA8627
					BOLD:ABA8628
	*Scatella obsoleta*	*CDC (2)*	1.25	2.5	BOLD:ABA7493
					BOLD:ABA7494
	*Scatophila signata*	*CDC (2)*	3.3	3.3	BOLD:ABA7651
					BOLD:ABA7652
Fanniidae	*Fannia postica*	*CDC (2)*	2.35	7.03	BOLD:ABW2012
					BOLD:ACG3518
Heleomyzidae	*Heleomyza serrata*	*CDC (2)*	0.37	3.54	BOLD:ABX8716
					BOLD:ACV1127
Lauxaniidae	*Minettia longipennis*	*CDC (2)*	0.96	1.45	BOLD:ACR0546
					BOLD:ACR0548
Limoniidae	*Chionea lutescens*	*CDC (2)*	1.1	1.1	BOLD:ABV5195
					BOLD:ADD1050
	*Euphylidorea meigenii*	*CDC (2)*	1.91	4.88	BOLD:ABV4905
					BOLD:ACU9122
Milichiidae	*Phyllomyza equitans*	*CDC (2)*	1.39	4.05	BOLD:ACB3455
					BOLD:ACD3072
Muscidae	*Helina evecta*	*CDC (3)*	1.83	4.27	BOLD:AAE3133
					BOLD:ACB3279
					BOLD:ADB5997
	*Mydaea humeralis*	*CDC (2)*	1.95	5.84	BOLD:AAE0058
					BOLD:ACD1934
Mycetophilidae	*Boletina dispecta*	*CDC (3)*	9.01	11.2	BOLD:AAY5579
					BOLD:AAY5580
					BOLD:AAY5581
	*Brevicornu griseicolle*	CDC(2)	9.06	13.6	BOLD:ACU9474
					BOLD:ABA1563
	*Brevicornu sericoma*	*CDC (2)*	1.99	4.58	BOLD:AAY6368
					BOLD:ABA1564
	*Phronia obtusa*	*CDC (2)*	0.83	1.18	BOLD:AAY8505
					BOLD:ACJ2989
	*Stigmatomeria crassicornis*	*CDC (2)*	0.56	1.86	BOLD:AAY6370
					BOLD:ACU7541
	*Zygomyia angusta*	*CDC (3)*	3.29	14.88	BOLD:AAY5526
					BOLD:AAY5527
					BOLD:ABW0168
	*Zygomyia valida*	*CDC (2)*	9.51	14.5	BOLD:AAY5526
					BOLD:ABW0168
Pallopteridae	*Toxoneura aff. modesta*	*CDC (2)*	3.41	5.13	BOLD:ACB4053
					BOLD:ACV1580
Phoridae	*Megaselia consetigera*	*CDC (2)*	0.65	2.63	BOLD:ACG2938
					BOLD:ACX1476
	*Megaselia glabrifrons*	*CDC (2)*	0.66	1.78	BOLD:ACG3433
					BOLD:ACI6910
	*Megaselia longicostalis*	*CDC (3)*	1.32	5.72	BOLD:AAG3263
					BOLD:ADA4916
					BOLD:AAG7025
	*Megaselia lutea*	*CDC (2)*	2.14	6.46	BOLD:AAG3351
					BOLD:ACG3608
	*Megaselia nigriceps*	*CDC (3)*	0.76	7.16	BOLD:AAG7022
					BOLD:AAY6384
					BOLD:ACF7950
	*Megaselia pulicaria complex*	*CDC (3)*	5.85	11.96	BOLD:AAL9073
					BOLD:AAP4698
					BOLD:AAU8534
	*Megaselia rufa*	*CDC (2)*	1.83	8.31	BOLD:ACD9573
					BOLD:ACD9606
	*Megaselia ruficornis*	*CDC (2)*	5.46	17.53	BOLD:ACF7708
					BOLD:ACG4585
	*Megaselia sepulchralis*	*CDC (2)*	2.27	4.27	BOLD:ACF7622
					BOLD:ACZ9853
	*Megaselia subpalpalis*	*CDC (2)*	1.05	2.17	BOLD:AAL9083
					BOLD:ACZ7449
	*Megaselia tarsella*	*CDC (3)*	0.45	5.61	BOLD:ACE0332
					BOLD:ACF7226
Psychodidae	*Psychoda nr. albipennis*	*CDC (2)*	1.55	3.45	BOLD:ABA0876
					BOLD:ACN5049
Rhinophoridae	*Rhinomorinia sarcophagina*	*CDC (2)*	0.75	1.78	BOLD:ACD9526
					BOLD:ACG3259
Sciaridae	*Bradysia brevispina*	*CDC (2)*	2.86	8.4	BOLD:ACE4845
					BOLD:ACI5443
	*Bradysia inusitata*	*CDC (2)*	6.61	6.61	BOLD:ACE7273
					BOLD:ACH4332
	*Bradysia praecox*	*CDC (2)*	1.09	2.35	BOLD:ACF3561
					BOLD:ACU9870
	*Bradysia regularis*	*CDC (2)*	0.1	1.67	BOLD:ACC1391
					BOLD:ACQ7807
	*Bradysia tilicola*	*CDC (2)*	2.87	6.03	BOLD:AAN6444
					BOLD:ACP0919
	*Bradysia trivittata*	*CDC (2)*	0.57	3.57	BOLD:AAH3947
					BOLD:ACB1143
	*Bradysiopsis vittata*	*CDC (2)*	2.24	4.62	BOLD:ACC1999
					BOLD:ACR0949
	*Corynoptera grothae*	*CDC (2)*	4.75	9.36	BOLD:ACK0158
					BOLD:ACO7236
	*Corynoptera luteofusca*	*CDC (2)*	8.16	11.8	BOLD:ACJ1951
					BOLD:ACQ8494
	*Corynoptera polana*	*CDC (2)*	1.95	3.81	BOLD:ACF6941
					BOLD:ACF7764
	*Corynoptera subtilis*	*CDC (2)*	2.91	6.26	BOLD:ACD5314
					BOLD:ACT9420
	*Corynoptera tetrachaeta*	*CDC (2)*	4.16	4.16	BOLD:ACG5327
					BOLD:ACL4032
	*Corynoptera tridentata*	*CDC (2)*	9.95	9.95	BOLD:ACJ1561
					BOLD:ACJ9791
	*Epidapus atomarius*	*CDC (2)*	0.07	3.98	BOLD:ACD4767
					BOLD:ACX3063
	*Leptosciarella fuscipalpa*	*CDC (2)*	5.24	9.24	BOLD:ACE2641
					BOLD:ACQ8733
	*Leptosciarella scutellata*	CDC (3)	4.84	7.98	BOLD:ACD6061
					BOLD:ACG4078
					BOLD:ACI9623
	*Pnyxiopsis degener*	*CDC (2)*	1.83	5.17	BOLD:ACE2293
					BOLD:ACF9729
	*Scatopsciara neglecta*	*CDC (2)*	0.53	1.78	BOLD:ACC7986
					BOLD:ACQ2637
	*Scatopsciara subciliata*	*CDC (2)*	1.93	4.32	BOLD:AAH4004
					BOLD:ACA8369
	*Sciara hemerobioides*	*CDC (2)*	4.1	4.1	BOLD:ACQ8933
					BOLD:ACR4627
	*Trichosia morio*	*CDC (2)*	0.78	3.99	BOLD:ACD5342
					BOLD:ACO9950
Simuliidae	*Simulium cryophilum*	*CDC (2)*	1.49	3.14	BOLD:ACU9243
					BOLD:AAU1818
Sphaeroceridae	*Opacifrons coxata*	*CDC (2)*	6.41	14	BOLD:ACP2618
					BOLD:ACP5793
	*Spelobia clunipes*	*CDC (2)*	2.89	6.93	BOLD:AAG7312
					BOLD:ACF9400
Syrphidae	*Cheilosia albipila*	*CDC (2)*	2.51	6.88	BOLD:AAW3610
					BOLD:AAZ1026
	*Cheilosia chrysocoma*	*CDC (2)*	3.69	3.69	BOLD:ABY6892
					BOLD:ACJ5068
	*Cheilosia derasa*	*CDC (2)*	0.58	3.47	BOLD:AAY9044
					BOLD:AAW3649
	*Cheilosia flavipes*	*CDC (2)*	8.79	8.79	BOLD:AAW3610
					BOLD:AAY9045
	*Cheilosia impressa*	*CDC (2)*	1.95	5.74	BOLD:AAW3651
					BOLD:AAW3615
	*Cheilosia lenis*	*CDC (2)*	3.85	7.86	BOLD:AAY8876
					BOLD:AAY8875
	*Cheilosia mutabilis*	*CDC (2)*	1.94	2.74	BOLD:AAY9746
					BOLD:AAY9747
	*Cheilosia personata*	*CDC (2)*	1.35	1.88	BOLD:ACH1700
					BOLD:ACX0819
	*Cheilosia proxima*	*CDC (3)*	3.28	6.91	BOLD:AAW3607
					BOLD:AAW3651
					BOLD:ABY8734
	*Cheilosia vernalis‐agg*.	*CDC (2)*	2.07	3.84	BOLD:ACF0974
					BOLD:ACJ5218
	*Eupeodes nitens*	*CDC (2)*	3.97	3.97	BOLD:AAB2384
					BOLD:ACH1529
	*Melanogaster nuda*	*CDC (2)*	0.81	2.44	BOLD:AAY8880
					BOLD:ACH5745
	*Merodon rufus*	*CDC (2)*	0.68	1.09	BOLD:ADI8358
					BOLD:AAQ1380
	*Paragus pecchiolii*	*CDC (2)*	0.96	4.86	BOLD:ABA3664
					BOLD:ACG8255
	*Parasyrphus punctulatus*	*CDC (2)*	1.11	2.65	BOLD:AAZ4514
					BOLD:ACG4772
	*Pipiza noctiluca*	*CDC (2)*	1.54	3.92	BOLD:AAL4100
					BOLD:ACG4983
	*Platycheirus albimanus*	*CDC (2)*	0.37	3.01	BOLD:AAL7898
					BOLD:ACJ4919
	*Sericomyia lappona*	*CDC (2)*	2.06	3.9	BOLD:AAB1553
					BOLD:ACH1641
Tabanidae	*Tabanus bromius*	*CDC (2)*	2.04	2.93	BOLD:AAF3864
					BOLD:ACJ5745
	*Tabanus glaucopis*		3.27	4.43	BOLD:AAF3858
					BOLD:AAF3859
Tachinidae	*Actia dubitata*	*CDC (2)*	2.36	2.36	BOLD:ACP3766
					BOLD:ACH1972
	*Bessa selecta*	*CDC (2)*	1.45	2.38	BOLD:ADK1760
					BOLD:AAW3422
	*Cyzenis albicans*	*CDC (2)*	1.18	2.18	BOLD:ACB0896
					BOLD:ACM9631
	*Kirbya moerens*	*CDC (2)*	1.22	1.86	BOLD:ACJ2730
					BOLD:ACB0261
	*Peribaea fissicornis*	*CDC (2)*	2.22	8.17	BOLD:ACH1961
					BOLD:ACJ2910
	*Phorinia aurifrons*	*CDC (2)*	3.76	11.2	BOLD:ADK4076
					BOLD:ACB0795

Appendix S1 provides species names, sample IDs, BIN assignment and collection information. All project data are available under the publicly accessible DOI: http://dx.doi.org/10.5883/DS-DIPBFGBL.

### 3.2 Performance of the reference library for metabarcoding of Malaise trap samples

**Figure 2:**
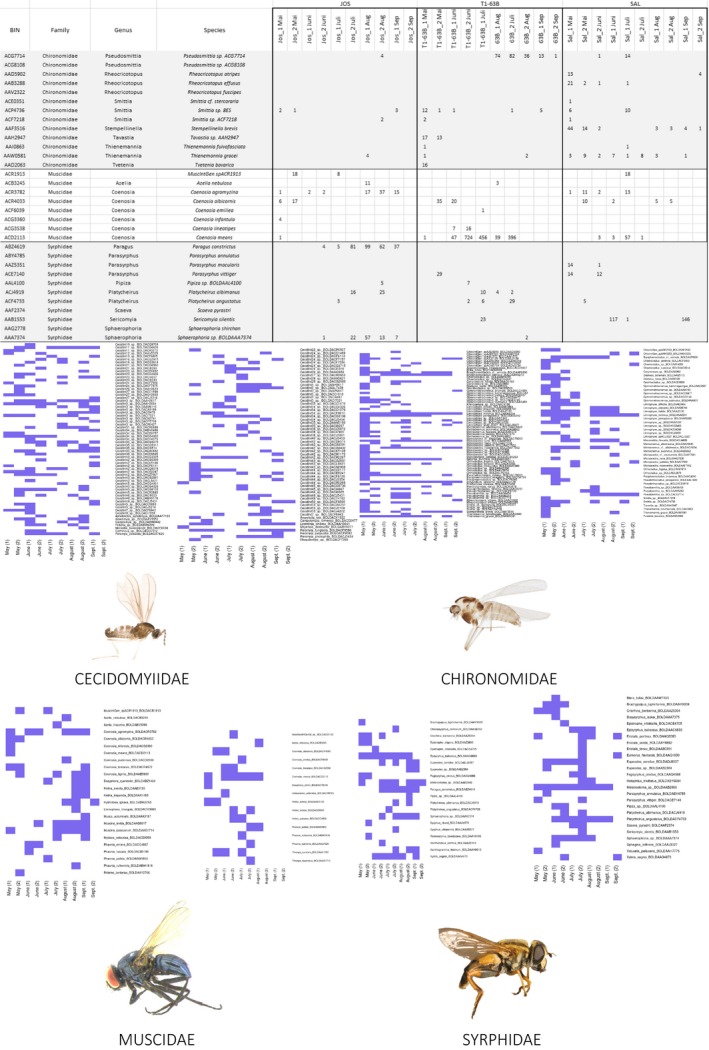
Examples from the metabarcoding results. Presence–absence overviews for three sample sites (Jos, T1‐63B and SAL) and illustrative examples for the families Cecidomyiidae, Chironomidae, Muscidae and Syrphidae [Colour figure can be viewed at http://www.wileyonlinelibrary.com]

Among the 90 Malaise trap samples from the Bavarian Forest National Park (L. A. Hardulak et al. in prep.), metabarcoding revealed 1,735 dipteran OTUs, comprising 536,376 reads: 5,960 average reads per sample, matching at 97% or higher to a taxon in the DNA barcode library downloaded from BOLD (average read count was 6,928 per sample with a total of 2,809 OTUs matched to the Diptera database with ≥90%). Multiple OTU matches to a single BIN were merged. Using the Diptera data, we identified a total of 1,403 BINs including representatives of 71 families (1,385 species) within the metabarcoding data set (Appendix S2). Almost one‐third (498/1403) of these BINs belonged to “dark taxa.” Figure 2 illustrates examples of presence/absence overviews for the families Muscidae, Cecidomyiidae, Chironomidae and Syrphidae for selected Malaise trap sites.

**Figure 3:**
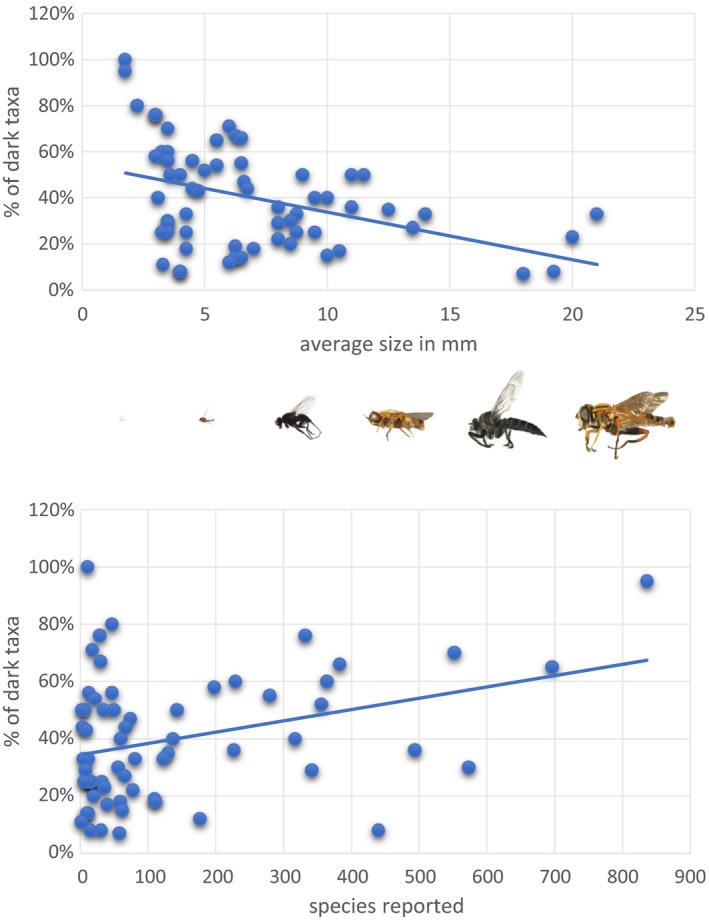
Illustration of the relationship between the percentage of “dark taxa” and average body size (mm), and in number of species reported for a family [Colour figure can be viewed at http://www.wileyonlinelibrary.com]

Among families containing “dark taxa,” the percentage of unnamed taxa was inversely correlated with body size (*r* = −0.41, *p* = 0.0004) and positively with numbers of species reported from Germany (r = 0.33, *p* = 0.0037) (Figure 3; Appendix S4).

## 4 DISCUSSION

This study summarizes the results of a DNA barcoding campaign on German Diptera, work based on the characterization of 45,040 specimens. The resultant DNA barcode reference library included records for 5,200 BINs (2,453 named species comprising 2,500 BINs plus 2,700 unnamed BINs) belonging to 88 families, covering ~ 50% of the Diptera fauna reported for Germany (Schumann, 2002, 2004, 2010; Schumann et al., 1999). Until now, most of these families, especially some of the most diverse, have been taxonomically inaccessible because of the lack of specialists. By contrast, within just a few years, this study provided an interim taxonomic identification system for half of the German Diptera fauna. Although half these species still lack a Linnean name, their BIN assignments are useful “taxonomic handles” for work in ecology, conservation biology and other biodiversity research (see Geiger, Moriniere, et al., 2016). The study demonstrates the efficiency of DNA barcoding in the identification of Central European Diptera, reinforcing the results of earlier studies. DNA barcode coverage was nearly complete for many species‐poor families (e.g., Megamerinidae, Opetiidae, Phaeomyiidae) known from Germany and the incidence of “dark taxa” in these families was low. Overall, there was a strong inverse relationship between the number of “dark taxa” and average body size: the smaller the average body size of a family, the higher the ratio of “dark taxa” (Figure 3). Among families with the smallest body sizes, our results suggest a higher incidence of cryptic diversity and overlooked species, indicating the number of dipteran species in Germany is likely to be much higher than previously recognized. Among families, such as the “Iteaphila group” (Empidoidea; see Meyer & Stark, 2015), Milichiidae and Trichoceridae, DNA barcoding indicates unexpectedly high levels of diversity as their BIN count is substantially higher than the number of species known from Germany (Schumann et al., 1999). The Cecidomyiidae represent the most impressive example, as we encountered 930 BINs while only 886 species are known from Germany (Table 1; Jaschhof, 2009; Schumann et al., 1999). As such, they represent by far the largest family of Diptera in the studied area. When compared with the other families in Figure 2b, it is clear that the Cecidomyiidae show a lower average interspecific variation, indicating an increased evolutionary rate. As already proposed by Hebert et al. (2016), the extraordinary species—or BIN number—might be linked to their unusual mode of reproduction, namely haplodiploidy. Here, paternally inherited genomes of diploid males are inactivated during embryogenesis (Normark, 2003). The phenomenon of haplodiploidy is known from Hymenoptera (Branstetter et al., 2018; Hansson & Schmidt, 2018) another group known to be rate accelerated, but it is largely unstudied throughout Diptera. Despite the need for more study, we conclude the true diversity of Diptera in Germany, Europe and the world has been seriously underestimated, a conclusion reached in several other studies (Erwin, 1982; Hebert et al., 2016; May, 1988; Ødegaard, 2000).

Within the metabarcoded Malaise trap samples collected over just one season in one region of Germany, we identified 1,735 OTUs with a sequence identity higher than 97% to a dipteran record. This result indicates that metabarcode analysis of bulk samples will be a valuable approach for assessing the diversity of Diptera in Germany (Appendix S2). Variation in overall biodiversity between sampling sites as well as annual phenologies of certain taxa can easily be visualized using presence–absence maps (Figure 2). This will be a useful feature for comparison of large data sets and for monitoring beneficial or pest insects (L. A. Hardulak et al. in preparation). Although a third of the OTUs within the metabarcoding data set could not be assigned to a Linnean species, interim names, such as BIN assignments, make it possible to compare sampling sites. OTUs with lower sequence similarities (<97%) to known taxa can be used to track “dark taxa,” those species missing from the reference sequence library. Although such taxa may only be assigned to a family or genus, their records are still valuable for evaluating differences between samples from various environments or sites. At present, dipteran species, although overall present in very high numbers, are extremely under‐represented within environmental assessments in Germany: ~2,000 species from 11 families (Asilidae, Atelestidae, Ceratopogonidae, Chaoboridae, Dixidae, Dolichopodidae, Empididae, Hybotidae, Psychodidae, Syrphidae, Thaumaleidae) are included in the German red list (Gruttke et al., 2016), but not a single dipteran species is listed among the ~1,000 species being protected according to the European Flora‐Fauna‐Habitat directive (Council Directive 92/43/EEC on the Conservation of natural habitats and of wild fauna and flora, 1992), which ensures the conservation of a wide range of rare, threatened or endemic animal and plant species in Europe. The present study is a first step to permit the proper evaluation of the status of dipterans and the potential designation of some species as targets for conservation action.

Previous studies have shown the great potential of metabarcoding for biotic assessments in various contexts, including Malaise trap surveys (Morinière et al., 2016), biosurveillance of invasive and pest species (Ashfaq & Hebert, 2016; L. A. Hardulak et al. in prep), macrozoobenthos sampling for assessing water and stream health (Elbrecht & Leese, 2015; Serrana, Miyake, Gamboa, & Watanabe, 2018), faeces analyses for dietary inference (De Barba et al., 2014; Hawlitschek, Fernández‐González, Balmori‐de la Puente, & Castresana, 2018), species identification for forensic entomology (Chimeno et al., 2018) and for soil biology (Oliverio, Gan, Wickings, & Fierer, 2018). This approach combines the advantages of DNA barcoding, namely the capacity to identify any life stage, body fragment or even trace DNA in the environment, with the ability of high‐throughput sequencers to analyse millions of DNA fragments and thousands of specimens at a time. The application of this technology to biodiversity assessments will certainly enable species surveys at larger scales, shorter time and lower costs compared with classical morphological approaches (Douglas et al., 2012; Hajibabaei et al., 2011; Ji et al., 2013; Taberlet, Coissac, Pompanon, Brochmann, & Willerslev, 2012). The ability to upscale biomonitoring projects is crucial, as is the need to generate biodiversity data fast and with less dependence on often unavailable taxonomic experts. Additionally, data generated by ongoing metabarcoding studies, such as from annual national biomonitoring projects, can be combined and reanalysed, producing recursively more comprehensive species lists, when new reference sequences become available or when taxonomic annotations have been improved. While biomonitoring studies have traditionally employed small subsets of indicator species, metabarcoding will enable comprehensive assessments of biodiversity because even “dark taxa” can be tracked. Furthermore, metabarcoding can enhance the ability to rapidly assess biodiversity patterns to identify regions that are of most significance for conservation.

Although this project aimed to develop a comprehensive DNA barcode library, resource constraints meant that only half the specimens sorted to a family or better taxonomy could be analysed. It is certain that many species and genera currently absent from the reference library remain within this sorted material, making the remaining samples a valuable resource for future extension of the reference library. Our work has also highlighted the potential of DNA barcoding and metabarcoding to aid efforts to conserve the world's fauna. Because these technologies greatly enhance our ability to identify, and thus conserve, biodiversity, they should be pursued—vigorously. As our study has provided several thousands of voucher‐based DNA barcode records, we invite the global community of dipteran taxonomists to improve identifications for the many “dark taxa” encountered in our study by identifying these vouchers using reverse taxonomic approaches.

The present study represents an important component of a decade of work directed toward creating a comprehensive DNA barcode library for German animal species. Because Diptera represents the largest and taxonomically most challenging insect order, they have received less attention than other orders (e.g., Lepidoptera, Coleoptera, freshwater orders) with lower species richness and more taxonomic expertise. Our work on Diptera has not only confirmed that this order is extremely species‐rich, but also that several of its most diverse families include a large proportion of “dark taxa.” The present study represents a cornerstone for subsequent research on these unexplored groups of Diptera. This paper presents the results of one of the most comprehensive studies on DNA barcoding of Diptera, with a coverage of over 80% of German families. Due to the general lack of taxonomy in many groups of Diptera, only a fraction of the specimens could be identified to species level. Most specimens for the study were obtained from just three Malaise traps deployed as a component of the Global Malaise programme (see http://biodiversitygenomics.net/projects/gmp/). Voucher specimens are still being identified by external specialists, a process that is labour intensive and time consuming, especially for taxonomically challenging taxa.

Our study presents results from one of the most comprehensive DNA barcoding projects on Diptera, a megadiverse, and, almost certainly, most diverse insect order. Our results strongly support the conclusion that DNA barcoding will enable the discovery and identification of most dipteran taxa. Some cases of low interspecific variation were observed in the Syrphidae, Tachinidae and Calliphoridae where additional markers may be needed for species identification (Haarto & Ståhls, 2014; Nelson et al., 2012; Pohjoismäki et al., 2016; Whitworth et al., 2007). However, in most cases, there was congruence between BINs and species defined by traditional morphological methods, supporting the use of DNA barcoding as a species identification tool for Diptera. This conclusion and the finding that many of the species we encountered represent “dark taxa” indicates that DNA barcoding will speed the discovery of genetic entities that will eventually gain recognition as biological species. Our data release aims at making these results accessible to the scientific community through a public data portal so they will be available for taxonomic research, biodiversity studies and barcoding initiatives at national and international levels.

In summary, the application of DNA barcoding enabled a comprehensive assessment of German Diptera, including several highly diverse families, which would otherwise have been excluded due to a lack of taxonomic expertise. By selecting morphospecies from the pool of specimens collected by the year‐long deployment of Malaise traps in ecosystems ranging from alpine to lowland settings, we constructed a reference library for most dipteran families known from Germany. Due to the diversity of sampling sites, we encountered a wide range of taxa from microendemics to wide‐ranging generalists with varied seasonal phenologies. We emphasize that DNA barcoding and the resultant barcode reference libraries provide an easy, intuitive introduction to molecular genetics, an approach accessible to undergraduate students in a way that genome sequencing is not. Because DNA barcoding workflows have been implemented in many laboratories around the world and because current primer sets reliably generate amplicons, this method is ideal for educational purposes. Democratization of the method, the analytical tools and data through the BOLD database (Ratnasingham & Hebert, 2007) further facilitates its use in real world situations. The approach has the additional advantage of allowing students to not only work with “real organisms,” but also to solve long‐standing taxonomic puzzles. The latter work leads students to probe the historical literature, to regale in past expeditions in search of type locations or type material, and potentially to end the chase by describing a new species. However, it is critical that senior taxonomists and professors need to recognize these possibilities and encourage their students to embrace this approach as it offers such a clear solution to the taxonomic impediment.

Germany has a tradition of more than 250 years of entomological research, and the number of Diptera species recorded is the highest for any European country comprising almost half of the European fauna. Despite this long effort, knowledge of its Diptera fauna must be regarded as fragmentary. In accordance with the species accumulation curve presented by Pape (2009) for the British Isles, additional species were revealed from current collecting efforts for practically every species‐rich family. Recording “new” species is slowed by the lack of experts for many of these families as well as by the lack of up‐to‐date identification keys. A particularly important result of our study is that the estimated number of dipteran species in Germany is certainly much higher than formerly thought. High proportions of unrecorded species were evident for the Agromyzidae, Anthomyiidae, Cecidomyiidae, Ceratopogonidae, Chironomidae, Chloropidae, Phoridae, Sciaridae and Sphaeroceridae, and to a lesser extent for the Empidoidea, Limoniidae, Mycetophilidae and others. Further studies point to an enormous under‐estimation of the species diversity in the Cecidomyiidae (Borkent et al., 2018; Hebert et al., 2016). Although our data do not allow for an accurate projection for the size of the total species numbers, it seems quite likely that this single family contains thousands of unrecorded species in Germany.

## AUTHOR CONTRIBUTIONS

Obtained funding: G.H., W.W., A.H., P.D.N.H. Collected the samples: D.D., B.R. Conceived and designed the experiments: J.M., L.A.H., M.G., B.R. Analysed the data: J.M., L.A.H., M.F.G., L.R., B.R. Wrote the paper: J.M., L.A.H., S.S., M.B., D.D. Contributed (additions/corrections) to the manuscript: P.D.N.H., A.H., M.F.G., L.H., G.H.

5

**Table 3:** All cases of low intraspecific sequence variation at *COI*; cases of BIN sharing (BS)

Family	Species	BS rank	Mean intraspecific variation	Max intraspecific	BIN
Anthomyiidae	*Hylemya nigrimana*	*BS (2)*	0.34	0.52	BOLD:ABA6492
	*Hylemya vagans*		0.37	1.58	
Calliphoridae	*Calliphora loewi*	*BS (2)*	1.07	1.07	BOLD:AAB6579
	*Calliphora vicina*		0.84	2.59	
	*Lucilia caesar*	*BS (3)*	0.95	3.07	BOLD:AAA7470
	*Lucilia caesarillustris*		0.7	2.43	
	*Lucilia illustris*		N/A	0	
Dolichopodidae	*Medetera petrophiloides*	*BS (2)*	0.35	1.22	BOLD:ACA1124
	*Medetera truncorum*		N/A	0	
	*Sphyrotarsus argyrostomus*	*BS (2)*	0.91	1.37	BOLD:ADB6106
	*Sphyrotarsus hygrophilus*		N/A	0	
Empididae	*Kowarzia madicola*	*BS (2)*	0	0	BOLD:ACJ7236
	*Kowarzia tenella*		5.39	10.8	
	*Kowarzia barbatula*	*BS (2)*	4.8	11.3	BOLD:ACJ6935
	*Kowarzia tenella*		5.39	10.8	
Ephydridae	*Allotrichoma bezzii*	*BS (4)*	0.13	0.31	BOLD:ACF1575
	*Allotrichoma filiforme*		0.08	0.15	
	*Allotrichoma laterale*		6.44	6.44	
	*Allotrichoma schumanni*		0	0	
	*Ephydra macellaria*	*BS (3)*	N/A	0	BOLD:AAG2729
	*Ephydra murina*		N/A	0	
	*Ephydra riparia*		2.83	2.83	
	*Hydrellia nigricans*	*BS (2)*	0.23	0.31	BOLD:ABA8624
	*Hydrellia subalbiceps*		0.31	0.46	
	*Notiphila cinerea*	*BS (2)*	0.26	0.46	BOLD:ABA7513
	*Notiphila graecula*		0	0	
	*Notiphila riparia*	*BS (2)*	0.16	0.35	BOLD:AAX5585
	*Notiphila subnigra*		0.41	0.62	
	*Philygria flavipes*	*BS (2)*	1.19	2.03	BOLD:ACK3229
	*Philygria punctatonervosa*		0.15	0.15	
	*Psilopa compta*	*BS (2)*	0.08	0.16	BOLD:AAG6948
	*Psilopa nitidula*		0.38	0.77	
Iteaphila‐group	*Anthepiscopus indet*.	*BS (2)*	0.14	0.48	BOLD:ACD9492
	*Anthepiscopus* sp. 1		11.3	11.3	
	*Anthepiscopus* sp. 1	*BS (2)*	11.3	11.3	BOLD:ACJ7111
	*Anthepiscopus* sp. 4		0.91	1.58	
	*Iteaphila* sp. 1	*BS (2)*	0.07	0.15	BOLD:ACD3033
	*Iteaphila* sp. 2		4.49	9.77	
Lonchopteridae	*Lonchoptera lutea*	*BS (2)*	0.39	1.09	BOLD:ABX0277
	*Lonchoptera nitidifrons*		N/A	0	
Muscidae	*Hydrotaea dentipes*	*BS (2)*	2.15	9.78	BOLD:AAZ9882
	*Hydrotaea similis*		0	0	
Mycetophilidae	*Boletina gripha*	*BS (2)*	0.52	0.9	BOLD:AAF6783
	*Boletina groenlandica*		N/A	0	
	*Mycetophila distigma*	*BS (2)*	N/A	0	BOLD:AAY8340
	*Mycetophila flava*		0.19	0.19	
	*Zygomyia angusta*	*BS (2)*	4.6	15.4	BOLD:AAY5526
	*Zygomyia valida*		14.5	14.5	
	*Zygomyia angusta*	*BS (2)*	4.6	15.4	BOLD:ABW0168
	*Zygomyia valida*		14.5	14.5	
Phoridae	*Triphleba bicornuta*	*BS (2)*	N/A	0	BOLD:ACF0365
	*Triphleba* sp. BOLD:ACF0365		0.66	1.22	
Sarcophagidae	*Sarcophaga depressifrons*	*BS (2)*	0	0	BOLD:ABV4597
	*Sarcophaga haemorrhoa*		0.47	0.7	
Simuliidae	*Simulium balcanicum*	*BS (2)*	N/A	0	BOLD:AAM4036
	*Simulium equinum*		1.59	2.66	
Syrphidae	*Baccha elongata*	*BS (6)*	N/A	0	BOLD:ABA3006
	*Baccha elongata s.s*.		0	0	
	*Baccha obscuripennis*		1.23	2.02	
	*Baccha* sp. BOLDABA3006		N/A	0	
	*Brachypalpus laphriformis*		0.56	1.54	BOLD:AAY9039
	*Brachypalpus valgus*		N/A	0	
	*Cheilosia albipila*	*BS (2)*	2.51	6.88	BOLD:AAW3610
	*Cheilosia flavipes*		8.79	8.79	
	*Cheilosia barbata*	*BS (3)*	0.1	0.3	BOLD:AAW3615
	*Cheilosia impressa*		1.95	5.74	
	*Cheilosia* sp. BOLDAAW3615		0	0	
	*Cheilosia chloris*	*BS (8)*	0.57	1.42	BOLD:ACF0974
	*Cheilosia chlorus*		0.12	0.18	
	*Cheilosia chlorus‐group*		N/A	0	
	*Cheilosia fraterna*		0.55	0.87	
	*Cheilosia melanura*		0.06	0.2	
	*Cheilosia ruficollis*		N/A	0	
	*Cheilosia* sp. BOLDACF0974		0.47	0.71	
	*Cheilosia vernalis‐agg*.		2.07	3.84	
	*Cheilosia crassiseta*	*BS (6)*	N/A	0	BOLD:AAW3647
	*Cheilosia impudens*		N/A	0	
	*Cheilosia nigripes*		N/A	0	
	*Cheilosia* sp. BIOUG17085‐G07		0.75	1.94	
	*Cheilosia aff. grisella*		N/A	0	
	*Cheilosia antiqua*		N/A	0	
	*Cheilosia faucis*	*BS (2)*	0.7	0.88	BOLD:AAY8874
	*Cheilosia nivalis*		0	0	
	*Cheilosia grisella*	*BS (2)*	0.18	0.18	BOLD:AAW3619
	*Cheilosia pubera*		0.49	0.87	
	*Cheilosia canicularis*	*BS (2)*	0.08	0.38	BOLD:ACI2500
	*Cheilosia montana*		N/A	0	
	*Cheilosia carbonaria*	*BS (2)*	0.37	0.37	BOLD:AAY8876
	*Cheilosia lenis*		3.85	7.86	
	*Chrysotoxum bicinctum*	*BS (2)*	0.86	2	BOLD:AAJ0967
	*Chrysotoxum festivum*		0	0	
	*Dasysyrphus hilaris*	*BS (3)*	0.35	0.52	BOLD:AAA7375
	*Dasysyrphus laskai*		0.3	0.3	
	*Dasysyrphus venustus*		N/A	0	
	*Dasysyrphus lenensis*	*BS (3)*	0.58	0.58	BOLD:AAB2865
	*Dasysyrphus pinastri*		1.25	2.1	
	*Dasysyrphus* sp. BOLDAAB2865		0.12	0.17	
	*Eupeodes bucculatus*	*BS (5)*	1.14	3.13	BOLD:AAB2384
	*Eupeodes nielseni*		0.15	0.37	
	*Eupeodes nitens*		3.97	3.97	
	*Eupeodes* sp. BOLDAAB2384		0.39	1.03	
	*Eupeodes luniger*		0.53	1.05	
	*Melanogaster aerosa*	*BS (2)*	N/A	0	BOLD:AAQ4015
	*Melanogaster hirtella*		0.26	0.7	
	*Melanostoma dubium*	*BS (7)*	0	0	BOLD:AAB2866
	*Melanostoma mellinum*		0.58	1.21	
	*Melanostoma mellinum‐agg*.		N/A	0	
	*Melanostoma scalare*		0.49	1.3	
	*Melanostoma* sp. A		0	0	
	*Melanostoma* sp. B		0.11	0.16	
	*Melanostoma* sp. BOLDAAB2866		0.63	2.69	
	*Merodon avidus*	*BS (2)*	N/A	0	BOLD:AAQ1379
	*Merodon avidus* B		0.55	1.03	
	*Paragus aff. haemorrhous*	*BS (5)*	N/A	0	BOLD:ABZ4619
	*Paragus constrictus*		N/A	0	
	*Paragus haemorrhous*		0.26	0.87	
	*Paragus* sp. BOLDABZ4619		0.07	0.37	
	*Paragus tibialis*		N/A	0	
	*Paragus majoranae*	*BS (2)*	0.87	0.87	BOLD:ABA3664
	*Paragus pecchiolii*		0.96	4.86	
	*Parasyrphus lineola*	*BS (2)*	0.19	0.39	BOLD:ACE7140
	*Parasyrphus vittiger*		0.63	1.44	
	*Pipiza bimaculata*	*BS (4)*	N/A	0	BOLD:AAL4100
	*Pipiza nocticula*		N/A	0	
	*Pipiza noctiluca‐agg*.		N/A	0	
	*Pipiza* sp. BOLDAAL4100		0.55	1.65	
	*Platycheirus angustatus*	*BS (3)*	0.84	2.02	BOLD:ACF4733
	*Platycheirus europaeus*		1.95	1.95	
	*Platycheirus* sp. BOLDACF4733		0.21	1.15	
	*Platycheirus clypeatus*	*BS (5)*	0.38	0.88	BOLD:AAA9506
	*Platycheirus fulviventris*		1.04	1.04	
	*Platycheirus occultus*		0.51	1.04	
	*Platycheirus perpallidus*		N/A	0	
	*Platycheirus* sp. BOLDAAA9506		0.9	2.03	
	*Platycheirus melanopsis*	*BS (2)*	0.25	0.62	BOLD:AAP0412
	*Platycheirus tatricus*		N/A	0	
	*Platycheirus nielseni*	*BS (3)*	0	0	BOLD:AAC6630
	*Platycheirus peltatus*		0.24	0.72	
	*Platycheirus peltatus‐group*		N/A	0	
	*Platycheirus scutatus*	*BS (3)*	0.05	0.19	BOLD:AAG4665
	*Platycheirus scutatus‐group*		0.44	0.71	
	*Platycheirus splendidus*		N/A	0	
	*Scaeva dignota*	*BS (2)*	N/A	0	BOLD:AAF2374
	*Scaeva pyrastri*		0.25	0.91	
	*Scaeva pyrastri*	*BS (2)*	0.25	0.91	BOLD:AAF2374
	*Scaeva dignota*		N/A	0	
	*Sericomyia lappona*	*BS (2)*	2.06	3.9	BOLD:AAB1553
	*Sericomyia silentis*		0.05	0.24	
	*Sphaerophoria bankowskae*	*BS (9)*	N/A	0	BOLD:AAA7374
	*Sphaerophoria infuscata*		0.24	0.38	
	*Sphaerophoria interrupta*		0	0	
	*Sphaerophoria interrupta‐group*		0.49	0.75	
	*Sphaerophoria philanthus*		N/A	0	
	*Sphaerophoria rueppellii*		N/A	0	
	*Sphaerophoria* sp. BOLDAAA7374		0.31	6.54	
	*Sphaerophoria taeniata*		N/A	0	
	*Sphaerophoria virgata*		N/A	0	
	*Sphegina montana*	*BS (2)*	N/A	0	BOLD:ABX4867
	*Sphegina sibirica*		0.4	0.41	
	*Temnostoma apiforme*	*BS (2)*	0.52	0.52	BOLD:AAV6543
	*Temnostoma meridionale*		0.35	0.52	
Stratiomyidae	*Beris geniculata*	*BS (2)*	N/A	0	BOLD:AAW3384
	*Beris morrisii*		0.48	1.47	
Tachinidae	*Lydella stabulans*	*BS (2)*	0.12	0.44	BOLD:AAP8653
	*Lydella thompsoni*		0.68	1.31	
	*Medina luctuosa*	*BS (3)*	1.35	1.35	BOLD:AAG6902
	*Medina melania*				

## DATA AVAILABILITY

All specimen data have been made publicly available within the BOLD workbench ‐ a DOI for the dataset has been added.
