# Supplementary material for: A DNA barcode library for 5,200 German flies and midges (Insecta: Diptera) and its implications for metabarcoding‐based biomonitoring
Source: Mol Ecol Resour. 2019 May 14;19(4):900–28. doi: 10.1111/1755-0998.13022 (PMC6851627; doi:10.1111/1755-0998.13022)
Supplement: Supplementary file 1 [file MEN-19-900-s001.html]

Javascript must be enabled to view this page.

magnitude
magnitudeUnassigned

German\_flies\_and\_midges

5370

5370

5370

5370

42

35

7

1

1

1

1

1

1

1

6

2

1

1

1

1

2

1

1

3

1

1

1

2

1

1

9

1

1

1

1

1

1

1

1

1

5

1

1

2

1

1

1

1

1

5

1

1

4

1

1

1

1

1

1

320

1

1

1

131

3

1

1

1

2

1

1

1

1

25

1

1

1

1

1

1

1

1

1

1

1

1

1

1

1

1

2

1

1

1

1

1

1

1

1

1

3

1

1

1

7

1

2

1

1

1

1

1

1

1
11

2

1

1

1

1

1

2

1

1

1

4

1

1

1

1

2

1

1

6

1

1

1

1

1

1

1

1

1

1

1

1

10

1

1

1

1

1

1

1

1

1

1

1

1

1

1

1

1

1

1

6

1

1

1

1

1

1

7

1

1

1

1

1

1

1

12

1

1

1

1

1

1

1

1

2

1

1

4

1

1

2

9

2

1

1

1

1

1

1

1

7

1

1

1

1

1

1

1

1

1

1

1

3

3

1

1

1

185

2

1

1

2

1

1

5

1

1

1

1

1

7

1

1

1

1

1

1

1

1

1

2

1

1

1

1

1

1

6

1

1

1

1

1

1

3

1

1

1

6

1

1

1

1

1

1

1

1

5

1

1

1

1

1

2

1

1

9

1

1

1

1

1

1

1

1

1

1

1

4

1

1

2

11

1

1

1

1

1

1

1

1

1

1

1

1

1

1

1

2

1

1

4

1

1

1

1

1

1

8

1

1

1

1

1

2

1

3

1

1

1

3

1

1

1

5

1

1

1

2

4

1

1

1

1

1

1

75

2

1

1

1

1

1

1

1

1

1

1

1

2

2

1

1

1

1

2

1

1

1

1

1

1

1

1

1

1

1

1

2

1

1

2

1

1

2

1

1

1

1

1

2

1

1

2

1

1

1

1

2

1

1

1

1

3

1

1

1

1

1

1

3

1

1

1

4

1

1

1

1

1

1

163

117

32

1

1

1

1

1

1

1

1

1

1

1

1

1

1

1

1

1

1

1

1

1

1

1

1

1

1

1

1

1

1

1

1

35

1

1

1

1

1

1

1

1

1

1

1

1

1

1

1

1

1

1

1

1

1

1

1

1

1

1

1

1

1

1

1

2

1

1

9

1

1

1

1

1

1

1

1

1

41

1

1

1

1

1

1

1

1

1

1

1

1

1

1

1

1

1

1

1

1

1

1

1

1

1

1

1

1

1

1

1

1

1

1

1

1

1

1

1

1

1

13

1

1

1

1

6

1

1

1

1

1

1

1

1

2

1

1

2

2

1

1

3

1

1

1

20

1

1

2

1

1

7

2

1

2

1

1

6

1

1

1

1

1

1

4

1

1

1

1

1

1

7

1

1

1

1

1

1

1

1

1

3

3

1

1

1

30

15

1

1

3

1

1

1

1

1

1

1

2

1

1

2

1

1

4

1

1

1

1

1

1

1

1

2

1

1

12

2

1

1

10

1

1

1

1

1

1

1

1

1

1

9

1

1

1

1

2

2

1

1

1

1

1

1

1

1

1

1

4

1

1

3

1

1

1

134
2

1

1

72

17

1

1

1

1

1

1

1

1

1

1

1

1

1

1

1

1

1

55

1

1

1

1

1

1

1

1

1

1

1

1

1

1

1

2

1

1

1

1

1

1

1

1

1

1

1

1

2

1

1

1

1

1

1

1

1

1

1

1

1

1

1

1

1

1

1

1

1

1

1

1

1

34

1

1

5

1

1

2

1

15

1

1

1

1

1

1

1

1

1

1

1

1

1

1

1

5

1

2

1

1

4

1

1

1

1

1

1

1

1

1

1

1

1

1

1

2

1

1

3

1

1

1

3

1

1

1

16

16

1

1

1

1

1

1

1

1

1

1

1

1

1

1

1

1

47

1

1

39

4

1

1

1

1

14

1

1

1

1

1

2

1

1

1

1

1

2

15

1

1

1

1

1

1

1

1

1

1

1

1

1

1

1

1

1

1

1

4

1

1

1

1

1

1

6

1

1

5

1

1

1

1

1

4

3

2

1

1

1

1

1

1

1

2

2

1

1

4

2

1

1

2

1

1

2

2

1

1

1

1

20

1

1

5

5

1

1

1

1

1

1

1

1

1

1

1

1

1

1

1

2

1

1

1

1

1

1

1

1

1

1

4

4

1

1

1

1

28

18

1

1

1

1

1

1

1

1

1

1

1

1

1

1

1

1

1
8

1

1

1

1

1

1

1

2

1

1

8

1

1

2

1

1

2

1

1

1

1

1

1

1

1

1

1

1

1

1

8

5

5

1

1

2

1

3

3

1

1

1

9

1

1

1

5

5

1

1

1

1

1

3

2

1

1

1

1

141

30

20

1

1

1

1

1

1

1

1

1

1

1

1

1

2

1

1

1

1

1

10

1

1

1

1

1

1

1

1

1

1

12

1

1

1

1

1

1

9

2

1

1

1

2

1

1

1

1

4

1

1

1

1

1

1

1

1

18

5

1

1

1

2

1

1

3

1

1

1

7

1

1

1

1

1

1

1

2

2

3

2

1

1

1

1

3

1

1

1

10

4

1

1

1

1

1

1

1

1

3

1

1

1

1

1

26

1

1

9

1

1

1

1

1

1

1

1

1

1

1

1

1

14

1

1

1

1

1

2

1

1

1

1

1

1

1

34

4

1

1

1

1

2

1

1

3

1

1

1

3

1

1

1

1

1

8

1

1

2

1

1

1

1

2

2

1

1

9

1

2

1

1

1

1

1

1

1

1

175

1

1

34

1

1

3

1

1

1

4

1

1

1

1

12

1

1

1

1

1

1

1

1

1

1

1

1

4

1

1

1

1

2

1

1

8

1

1

1

1

1

1

1

1

1

1

1

1

1

1

1

1

1

1

1

1

1

1

1

1

1

1

1

1

1

1

1

1

27

1

1

1

1

5

1

1

1

1

1

15

1

1

1

1

1

1

1

1

1

1

1

1

1

1

1

1

1

3

2

1

1

1

1

1

15

1

1

5

2

1

1

1

1

1

8

1

1

1

1

1

1

2

1

1

1

1

1

1

1

1

1

1

1

1

1

1

1

1

1

1

1

1

1

1

1

1

1

1

1

1

1

1

1

1

1

1

1

1

1

1

1

1

1

1

1

1

1

1

1

1

1

1

1

1

1

1

1

1

1

1

12

1

1

1

1

1

1

2

1

1

3

1

1

1

1

1

2

1

1

1

1

1

1

1

1

1

1

40

15

1

1

1

1

1

3

1

1

1

1

1

1

1

23

1

1

1

1

2

1

1

1

1

1

1

1

1

1

1

1

1

1

1

3

2

1

1

1

1

1
103

1

1

1

1

3

1

1

1

3

1

1

1

66

1

1

1

1

4

1

1

1

1

4

1

1

1

1

18

1

1

1

1

1

1

1

1

1

1

1

1

1

1

1

1

1

1

4

1

1

1

1

3

1

1

1

1

1

3

1

1

1

2

1

1

1

1

1

1

2

1

1

2

1

1

1

1

10

1

1

1

1

1

1

1

1

1

1

6

1

1

1

1

1

1

1

1

1

1

27

9
1

1

1

1

1

1

1

1

1

4

1

1

1

1

1

1

1

1

1

1

1

1

7

1

1

1

1

1

1

1

3

1

1

1

1

1

28

22

12

1

1

1

1

1

1

1

1

1

1

1

1

2

1

1

1

1

2

1

1

3

1

1

1

2

1

1

6

2

1

1

1

1

2

1

1

1

1

15

14

1

1

9

1

1

1

2

1

1

1

1

3

2

1

1

1

1

1

1

1

1

57
1

3

1

1

1

3

1

1

1

1

1

21

3

1

1

1

1

1

1

1

2

1

1

4

2

1

1

8

1

1

1

1

1

1

1

1

2

1

1

1

1

5

4

1

1

1

1

1

1

21

21

1

1

1

1

1

1

1

1

1

1

1

2

1

1

1

1

2

1

1

1

1

8

2

2

1

1

6

4

1

1

1

1

2

1

1

142

1

1

11

8

1

1

1

1

1

1

1

1

3

1

1

1

11

5

1

1

1

1

1

1

1

2

1

1

1

1

2

1

1

13

3

1

1

1

1

1

9

1

1

1

1

1

1

1

1

1

106

2

1

1

11

1

1

1

2

1

1

1

1

1

1

4

2

1

1

2

1

1

2

1

1

6

1

1

1

1

1

1

1

1

78

1

1

1

1

1

1

1

1

1

1

1

1

1

1

1

1

1

1

1

1

1

1

1

1

1

1

1

1

1

1

1

1

1

2

1

1

1

1

1

1

1

1

1

1

1

1

1

1

1

1

1

1

1

1

1

1

1

1

1

1

1

1

1

1

1

1

1

1

1

1

1

1

1

1

1

1

1

115
1

6

1

1

2

1

1

3

1

1

1

14

14

1

1

1

1

1

1

1

1

1

1

1

1

1

1

3

3

1

1

1

26

1

1

25

1

1

1

1

1

1

1

1

1

1

1

1

1

1

1

1

1

1

1

1

1

1

1

1

1

3

3

1

1

1

5

2

1

1

2

1

1

1

1

1

1

9

8

2

1

1

1

1

2

1

1

9

1

1

1

1

1

1

1

1

1

27

4

1

1

1

1

1

1

4

1

1

1

1

18

1

1

1

1

1

1

1

1

1

1

1

1

1

1

1

1

1

1

1

1

8

5

1

1

1

1

1

3

1

1

1

2

2

1

1

9

4

1

1

1

1

2

1

1

1

1

1

1

1

1

1

1

1

1

1

51

1

1

10

1

1

1

1

1

1

1

1

1

1

1

1

1

1

1

1

1

1

1

1

1

33

1

1

19

1

1

1

1

2

1

1

1

1

1

1

1

1

1

1

1

1

1

3

1

1

1

1

1

1

1

1

1

1

1

3

1

1

1

1

1

1

1

1

1

1

1

1

1

7

2

1

1

1

1

5

1

1

2

1

1

1

1

1

1

12

1

1

1

1

2

2

1

1

5

1

1

2

1

1

2

1

1

1

1

1

1

1

1

313

32

20

2

3

1

1

1

1

1

1

1

1

1

1

1

1

1

1

1

1

1

1

1

1

1

2

1

1

2

1

1

1

1

2

1

1

1

1

1

1

1

1

1

1

1

1

2

1

1

1

1

1

1

1

1

2

1

1

11

5

1

1

1

1

1

5

1

1

1

2

1

1

1

1

1

1

1

1

1

1

1

1

1

1

1

1

1

1

1

1

1

1

1

1

1

1

1

1

1

1

21

18

1

1

1

1

1

1

1

1

1

1

1

1

1

1

1

1

1

1

3

1

1

1

193

6

1

1

1

1

1

1

109

2

1

1

2

1

1

11

1

1

1

1

1

2

1

3

3

1

1

1

27

1

1

1

1

1

1

2

1

1

1

1

1

1

1

1

1

1

1

1

1

1

1

1

1

1

1

13

1

1

1

1

1

1

1

1

1

1

1

1

1

1

1

50

1

1

1

1

1

1

1

1

1

1

1

1

1

1

1

1

1

1

1

1

1

1

1

1

1

1

1

1

1

1

1

1

1

1

1

1

1

1

1

1

1

1

1

1

1

1

1

1

1

1

15

1

1

1

1

1

1

2

1

1

1

1

2

1

63

6

1

1

1

1

1

1

2

1

1

14

1

1

1

1

1

1

1

1

1

1

2

1

1

2

1

1

4

1

1

1

1

8

1

1

1

1

1

1

1

1

6

1

1

1

1

1

1

2

1

1

13

2

1

1

1

1

1

1

1

1

1

1

1

2

1

1

2

1

1

2

1

1

15

1

1

1

1

1

1

1

1

2

1

1

1

1

2

1

1

1

1

3

1

1

1

2

1

1

1

1

3

1

1

1

1

1

1

1

1

1

1

1

2

1

1

1

1

1

1

1

1

1

1

1

1

1

1

1

1

1

1

1

1

1

1

1

1

1

1

1

7

7

2

2

1

1

1

1

1

3

3

1

1

1

1

1

1

2

2

1

1

5

5

1

1

4

1

1

1

1

17

1

1

11

1

1

2

1

1

1

1

3

1

1

1

4

1

1

2

1

1

1

1

2

1

1

1

1

13

10

3

1

1

1

2

1

1

5

1

1

1

1

1

3

3

1

1

1

3

2

2

1

1

1

1

1

2

2

2

1

1

1

1

1

46

1

1

2

1

1

1

1

1

1

1

1

28

19

1

1

1

1

1

1

1

1

1

1

1

1

1

1

1

1

1

1

1

2

1

1

7

1

1

1

1

1

1

1

1

1

1

1

1

1

1

1

1

1

1

1

1

1

1

1

1

1

1

1

1

1

1

1

1

1

1

3

1

1

2

1

1

7

1
7

1

1

1

1

1

1

12

11

1

1

1

1

1

1

1

1

1

1

2

1

1

1

1

3

1

1

1

1

1

26

1

1

9

9

1

1

1

1

1

1

1

2

1

1

1

1

1

1

1

1

1

1

2

2

1

1

1

1

1

1

1

1

1

1

1

1

1

1

1

1

1

1

81

46

1

1

1

1

1

1

1

1

1

1

1

1

1

1

1

1

2

1

1

1
2

1

2

2

5

1

1

1

1

1

1

1

12

1

1

1

1

1

1

2

1

1

1

1

2

1

1

3

1

1

1

3

1

1

1

1

1

1

1

3

1

1

1

1

1

10

1

1

2

1

1

7

1

2

1

1

1

1

1

1

1

1

1

1

2

2

1

1

1

1

1

1

1

1

1

1

1

1

1

1

1

1

1

1

1

1

1

1

1

1

1

1

1

1

1

1

1

1

1

1

1

1

1

1

1

1

1

1

1

1

1

1

3

3

1

1

2

1

1

30

1

1

1

1

1

1

1

1

1

1

1

1

1

1

1

1

1

1

1

1

1

1

6

1

1

2

1

1

1

1

1

1

1

1

1

1

1

1

1

1

1

1

1

1

1

1

1

1

2

2

1

1

1

1

1

1

1

1

1

1

1

1

1

313

27

1

1

1

1

1

1

1

1

1

1

1

1

2

1

1

1

1

1

1

1

3

1

1

1

2

1

1

1

1

1

1

71

1

1

1

2

1

1

1

1

1

2

1

1

1

1

2

1

1

1

1

1

1

1

1

1

1

1

1

1

3

1

1

2

1

1

1

1

1

1

1

1

1

1

1

1

2

1

1

1

1

1

1

1

1

1

1

1

1

1

1

1

2

1

1

2

2

1

1

3

2

1

7

1

1

1

1

1

1

1

3

1

1

1

17

1

1

1

1

1

2

2

1

1

1

1

1

1

1

1

1

1

1

1

1

1

10

2

1

1

1

1

1

1

1

1

12

1

1

1

2

1

2

1

1

1

1

7

1

2

1

1

1

1

7

1

1

1

1

1

1

1

1

1

2

1

1

5

1

1

1

1

1

1

1

6

1

1

1

1

2

1

1

1

1

1

1

1

1

1

1

34

1

1

1

1

1

1

1

1

1

1

1

1

1

1

1

1

1

1

1

1

1

1

1

1

1

1

1

1

1

1

1

1

1

1

2

2

61

1

1

1

1

1

1

1

1

1

1

1

1

1

1

1

2

1

1

1

1

1

1

1

1

1

2

1

1

2

1

1

1

2

1

1

1

1

2

1

1

2

1

1

1

2

1

1

1

1

1

1

1

1

1

16

1

1

1

1

1

1

1

1

2

1

1

1

1

1

1

1

1

4

1

1

1

1

1

1

10

5

1

1

2

1

1

2

2

5

3

1

1

1

1

1

1

1

7

7

1

1

1

1

1

1

1

14

14

1

2

1

1

1

1

1

1

1

1

1

1

1

295

1

1

1

1

1

1

1

1

1

1

1

1

1

1

1

1

1

1

1

1

2

2

1

1

1

1

1

1

1

1

1

1

1

1

1

1

1

1

1

1

1

1

1

1

1

1

1

1

1

1

1

1

1

1

1

1

1

1

1

1

1

1

1

1

1

1

1

1

1

1

1

1

1

1

1

1

1

1

1

1

1

1

1

1

1

1

1

1

1

1

1

1

1

1

1

1

1

1

1

1

1

1

1

1

1

1

1

1

1

1

1

1

1

1

1

1

1

1

1

1

1

1

1

1

1

1

1

1

1

1

1

1

1

1

1

1

1

1

1

1

1

1

1

1

1

1

1

1

1

1

1

1

1

1

1

1

1

1

1

1

1

1

1

1

1

1

1

1

1

1

1

1

1

1

14

1

1

1

1

1

1

1

1

1

1

1

1

1

1

1

1

45

5

1

1

1

1

1

1

1

4

1

1

1

1

2

2

15

1

1

1

1

1

1

1

1

1

1

1

1

1

1

1

5

1

1

1

1

1

2

1

1

1
7

2

1

1

2

4

1

1

1

1

1

1

1

1

1

1

1

1

1

1

1

1

1

1

1

1

134

1

1

2

1

1

3

1

1

1

124

1

1

1

1

1

1

1

1

1

1

1

1

1

1

3

1

1

1

1

1

1

1

1

1

1

1

1

1

1

1

1

1

1

2

1

1

1

2

1

1

1

1

1

1

1

1

1

1

1

1

1

1

1

2

2

1

1

1

1

1

2

1

1

1

1

1

1

1

1

1

1

1

1

1

1

2

1

1

1

3

2

1

1

1

3

1

1

1

1

1

1

1

1

1

1

1

1

1

1

1

2

1

1

1

1

1

1

1

2

1

1

3

1

1

1

2

1

1

1

1

1

1

1

1

1

1

2

2

1

1

1

1

1
20

19

5

1

1

1

1

1

14

1

1

2

1

1

1

1

1

1

1

1

1

1

8

3

2

1

5

1

1

1

1

1

2

1

1

1

1

1

2

2

1

1

1

1

930
1

9

1

1

1

1

1

1

1

1

1

40

1

1

1

1

1

1

1

1

1

1

1

1

1

1

1

1

1

1

1

1

1

1

1

1

1

1

1

1

1

1

1

1

1

1

1

1

1

1

1

1

9

1

1

1

1

1

1

1

1

1

1

1

1

1

4

1

1

1

1

24

1

1

1

1

1

1

1

1

1

1

1

1

1

1

1

1

1

1

1

1

1

1

1

1

31

1

1

1

1

1

1

1

1

1

1

1

1

1

1

1

1

1

1

1

1

1

1

1

1

1

1

1

1

1

1

1

3

3

1

1

1

142

95

1

1

1

1

1

1

1

1

1

1

1

1

1

1

1

1

1

1

1

1

1

1

1

1

1

1

1

1

1

1

1

1

1

1

1

1

1

1

1

1

1

1

1

1

1

1

1

1

1

1

1

1

1

1

1

1

1

1

1

1

1

1

1

1

1

1

1

1

1

1

1

1

1

1

1

1

1

1

1

1

1

1

1

1

1

1

1

1

1

1

1

1

1

1

1

8

1

1

1

1

1

1

1

1

6

1

1

1

1

1

1

2

1

1

1

1

2

1

1

1

1

1

1

3

1

1

1

3
1

1

1

1

1

8

1

1

1

1

1

1

1

1

2

1

1

3

1

1

1

4

1

1

1

1

2

1

1

1

1

3

1

1

1

8

1

1

1

1

1

1

1

1

28

1

1

1

1

1

1

1

1

1

1

1

1

1

1

1

1

1

1

1

1

1

1

1

1

1

1

1

1

27

1

1

1

1

1

1

1

1

1

1

1

1

1

1

1

1

1

1

1

1

1

1

1

1

1

1

1

10

1

1

1

1

1

1

1

1

1

1

8

1

1

1

1

1

1

1

1

3

1

1

1

9

1

1

1

1

1

1

1

1

1

2

1

1

5

1

1

1

1

1

21

1

1

1

1

1

1

2

1

1

1

1

1

1

1

1

1

1

1

1

1

9

1

1

1

1

6

1

1

1

1

1

1

1

1

24

2

1

1

2

1

1

6

1

1

1

1

1

1

1

1

12

1

1

1

1

1

1

1

1

1

1

1

1

1

1

1

1

41

1

1

1

1

1

1

1

1

1

1

1

1

1

1

1

1

1

1

1

1

1

1

1

1

1

1

1

1

1

1

1

1

1

1

1

1

1

1

1

1

1

49

1

1

1

1

1

1

1

1

1

1

1

1

1

1

1

1

1

1

1

1

1

1

1

1

1

1

1

1

1

1

1

1

1

1

1

1

1

1

1

1

1

1

1

1

1

1

1

1

1

2

1

1

5

1

1

1

1

1

11

1

1

1

1

1

1

1

1

1

1

1

5

1

1

1

1

1

18

1

1

1

1

1

1

1

1

1

1

1

1

1

1

1

1

1

1

8

1

1

1

1

1

1

1

1

2

1

1

31

1

1

1

1

1

1

1

1

1

1

1

1

1

1

1

1

1

1

1

1

1

1

1

1

1

1

1

1

1

1

1

25

1

1

1

1

1

1

1

1

1

1

1

1

1

1

1

1

1

1

1

1

1

1

1

1

1

10

1

1

1

1

1

1

1

1

1

1

1

1

64

1

1

1

1

1

1

1

1

1

1

1

1

1

1

1

1

1

1

1

1

1

1

1

1

1

1

1

1

1

1

1

1

1

1

1

1

1

1

1

1

1

1

1

1

1

1

1

1

1

1

1

1

1

1

2

1

1

1

1

1

1

1

1

2

1

1

4

1

1

1

1

5

1

1

1

1

1

48

1

1

1

1

1

1

1

1

1

1

1

1

1

1

1

1

1

1

1

1

1

1

1

1

1

1

1

1

1

1

1

1

1

1

1

1

1

1

1

1

1

1

1

1

1

1

1

1

1

1

59

1

1

1

1

1

1

1

1

1

1

1

1

1

1

1

1

1

1

1

1

1

1

1

1

1

1

1

1

1

1

1

1

1

1

1

1

1

1

1

1

1

1

1

1

1

1

1

1

1

1

1

1

1

1

1

1

1

1

1

8

1

1

1

1

1

1

1

1

8

1

1

1

1

1

1

1

1

1

1

42

1

1

1

1

1

1

1

1

1

1

1

1

1

1

1

1

1

1

1

1

1

1

1

1

1

1

1

1

1

1

1

1

1

1

1

1

1

1

1

1

1

1

2

1

1

20

1

1

1

1

1

1

1

1

1

1

1

1

1

1

1

1

1

1

1

1

6

1

1

1

1

1

1

9

1

1

1

1

1

1

1

1

1

5

1

1

1

1

1

7

1

1

1

1

1

1

1

1

2

1

1

1

1

2

1

1

4

1

1

1

1

1

1

5

1

1

1

1

1

3
1

1

1

1

1

461

1

1

19

2

1

1

1

1

1

1

1

1

1

1

1

1

1

1

1

1

2

1

1

1

1

7

1

1

1

1

1

1

1

308

35

1

1

1

1

1

1

1

1

1

1

1

1

1

1

1

1

1

1

1

1

1

1

2

1

1

1

1

1

1

1

1

1

1

1

8

1

1

1

1

1

1

1

1

1

1

5

1

1

1

1

1

13

1

1

1

1

1

1

1

1

1

1

1

1

1

2

2

5

1

1

1

1

1

6

1

1

1

1

1

1

2

2

1

1

16

1

1

1

1

4

3

1

1

1

1

1

5

1

1

1

1

1

2

1

1

44

1

1

1

1

1

1

1

1

1

1

1

1

3

1

1

1

1

1

1

1

1

1

1

2

1

1

2

1

1

1

1

2

1

1

1

1

1

1

1

1

1

9

1

1

1

1

1

1

1

1

1

17

1

1

1

1

1

1

1

1

1

1

1

1

1

1

1

1

1

2

1

1

2

1

1

1

1

1

1

4

1

1

1

1

1

1

15

1

1

1

1

1

1

1

1

1

1

1

2

1

1

14

1

1

1

1

1

1

1

1

1

1

1

1

1

1

13

1

1

1

1

1

1

1

1

1

1

1

1

1

10

1

1

2

1

1

1

1

1

1

28

1

1

1

3

1

1

1

1

1

2

1

1

1

1

1

1

1

1

1

1

1

1

1

1

1

2

1

1

1

1

8

1

1

1

1

2

1

1

4

1

1

1

1

1

1

4

1

1

1

1

7

1

1

1

1

1

1

1

4

1

1

1

1

5

1

1

1

1

1

2

1

1

3

1

1

1

2

1

1

1

1

1

1

1

1

2

1

1

2

1

1

1

1

1

1

1

1

1

1

1

1

1

1

2

1

1

1

1

4

1

1

1

1

1

1

10

2

1

1

7

1

1

1

1

1

1

1

1

1

2

2

1

1

3

1

1

1

1

1

1

1

1

1

1

1

74

2

1

1

22

1

1

1

1

1

1

1

1

1

1

1

1

1

1

1

1

1

1

1

1

1

1

1

1

1

1

2

1

1

2

1

1

3

1

1

1

2

2

1

1

2

1

1

5

1

1

1

1

1

1

1

2

1

1

5

1

1

1

1

1

3

1

1

1

9

9

1

1

1

1

1

1

1

1

1

1

1

7

1

1

1

1

2

1

2

1

1

1

1

3

1

1

1

1

1

1

18

1

1

1

1

1

1

1

2

1

1

1

1

1

1

1

2

1

1

1

1

223
1

1

1

1

1

1

1

1

1

1

1

1

1

1

1

1

1

1

1

58

1

1

3

1

1

1

4

1

1

1

1

1

1

1

1

4

1

1

1

1

5

1

1

2

1

1

1

5

1

1

1

1

1

1

1

1

1

2

1

1

2

1

1

7

1

1

2

2

1

3

1

1

1

2

1

1

2

1

1

1

1

1

1

1

1

1

1

4

1

1

1

1

4

2

1

1

1

1

1

1

1

1

1

1

1

1

1

1

1

1

1

1

1

1

1

1

11

1

1

1

1

1

1

1

1

1

1

1

4

2

1

1

2

1

1

1

1

1

1

1

1

1

1

1

1

56

2

1

1

1

1

1

1

1

1

1

1

1

1

1

1

3

1

1

1

1

1

1

1

2

1

1

1

1

2

1

1

1

1

1

1

1
5

1

1

1

1

1

1

2

1

1

1

1

2

2

2

1

1

1

1

1

1

1

1

1

1

1

1

3

1

2

1

1

1

1

1

1

1

1

2

1

1

2

2

1

1

1

1

1

1

1

1

1

1

1

1

1

1

1

1

1

1

1

1

1

1

1

1

1

1

1

1

1

1

1

1

1

1

1

1

1

1

1

1

27

1

1

3

1

1

1

1

1

1

1

1

1

2

1

1

2

1

1

1

1

3

1

2

2

1

1

1

1

2

1

1

1

1

2

1

1

2

2

1

1

1

1

1

1

1

1

2

2

17

1

1

1

1

1

1

3

1

1

1

1

1

6

1

1

1

1

1

1

1

1

1

1

1

1

1

1

1

1

1

1

1

1

1

1

1

1

1

1

1

1

1

1

1

1

218

1

1

50

9

1

1

1

1

1

1

1

1

1

23

1

1

1

1

1

1

1

1

1

1

1

1

1

1

1

1

1

1

1

1

1

1

1

18

1

1

1

1

1

1

1

1

1

1

1

1

1

1

1

2

1

165

19

1

1

1

1

1

1

1

1

1

1

1

1

1

1

1

1

1

1

1

2

1

1

3

1

1

1

6

1

1

2

1

1

6

1

1

1

1

1

1

7

1

1

1

1

1

1

1

1

1

27

1

1

1

1

1

1

1

1

1

1

1

1

1

1

1

1

1

1

1

1

1

1

1

1

1

1

1

1

1

31

1

1

1

1

1

1

1

1

1

1

1

1

1

1

1

1

1

1

1

1

1

1

1

1

1

1

1

1

1

1

1

7

1

1

1

1

1

1

1

3

1

1

1

52

1

1

1

1

1

1

1

1

2

1

1

1

1

1

1

1

1

1

1

1

1

1

1

1

1

2

1

1

1

1

1

1

1

1

1

1

1

1

1

1

1

1

1

1

1

1

1

1

1

1

1

1

1

1

7

1

1

1

2

1

1

4

3

1

1

1

1

1

4

1

1

3

3

1

1

1

1
187

1

1

57

4

1

1

1

1

6

1

1

1

1

1

1

2

1

1

3

1

1

1

6

1

1

1

1

1

1

35

1

1

1

1

1

1

2

1

1

1

1

1

1

1

2

1

1

1

1

1

1

1

1

2

1

1

1

1

1

1

1

1

1

1

128

2

1

1

2

1

1

4

1

2

1

8

2

1

1

1

2

1

5
1

1

1

1

1

22

1

1

1

1

1

1

1

1

1

1

1

1

1

1

1

1

1

2

1

1

1

2

1

1

3

1

1

1

14

2

1

1

1

1

1

1

1

1

1

1

1

1

35

1

1

1

1

1

1

1

1

1

1

1

1

1

1

1

1

1

1

1

1

1

1

1

1

1

1

1

1

1

1

1

1

1

1

1

6

1

1

1

1

1

1

3

1

1

1

5

1

1

1

1

1

8

1

1

1

1

1

1

1

1

2

1

1

3

1

1

1

4

1

1

1

1

1

1

1

48

2

1

1

46

43

1

1

1

1

1

2

1

1

1

2

1

1

1

1

1

1

1

1

2

1

2

1

1

1

1

1

1

1

1

1

1

1

1

1

1

1

1

1

1

3

1

1

1

2

2

1

1

1

1

96
1

1

1

8

1

1

1

1

1

1

1

1

2

1

1

11

1

1

1

1

1

1

3

1

1

1

3

1

2

1

1

1

1

33

1

1

8

1

1

1

1

1

1

2

2

1

1

2

1

1

1

1

8

1

1

1

1

1

1

1

1

1

1

3

1

2

1

1

5

1

1

1

1

1

1

1

27

3

1

1

1

1

1

1

1

4

1

1

1

1

1

1

9

1

1

1

1

1

1

1

1

1

1

1

1

1

6

1

1

1

1

1

1

4

1

1

1

1

1

1

1

1

2

1

1

1

4

1

1

1

1

19

16

2

1

1

1

1

8

1

1

1

1

1

2

1

1

1

1

1

1

1

2

1

1

3

1

1

1

13

1

1

1

1

1

1

1

1

1

1

1

1

1

1

1

1

1

1

1

1

1

1

1

1

1

1

52

1

1

5

2

1

1

2

1

1

1

1

1

1

1

1

1

1

4

1

1

2

2

1

1

32

1

1

26

1

1

1

1

1

2

1

1

1

1

1

2

1

1

1

1

1

1

1

1

1

1

1

1

4

1

1

1

1

1

1

1

1

1

1

1

1

1

1

1

1

1

1

1

1

1

18

2

2

1

1

4

1

1

1

1

1

1

1

1

1

1

1

1

1

4

1

1

3

1

1

1

3

3

1

1

1

1

1

1

1

1

1

1

2

2

1

1

16

14

1

1

1

1

1

1

10

1

1

1

1

1

1

1

1

1

1

1

1

2

2

1

1

17

17

8

1

1

1

1

1

1

1

1

9

1

1

1

1

1

1

1

1

1

12

12

9

1

1

2

1

1

2

1

3

1

1

1

6

1

1

1

5

5

1

1

1

1

1

42

22

1

1

11

2

1

1

1

1

1

1

1

1

1

1

1

6

1

1

1

1

1

1

3

1

1

1

1

1

1

9

9
1

1

1

1

1

1

1

1

1

1

1

1

5

2

1

1

1

1

1

1

1

1

4

1

1

3

1

2

22

7

1

1

5

1

1

1

1

1

1

1

1

1

1

3

1

1

2

1

1

2

1

1

1

1

5

1

1

3

1

1

1

1

1

1

1

1

1

1

1

1

1

1

26

23

4

1

1

1

1

1

1

1

1

4

1

1

1

1

1

1

1

1

3

1

1

1

4

2

1

1

1

1

2

1

1

1

1

1

1

2

1

1
