# Supplementary material for: A DNA barcode library for 5,200 German flies and midges (Insecta: Diptera) and its implications for metabarcoding‐based biomonitoring
Source: Mol Ecol Resour. 2019 May 14;19(4):900–28. doi: 10.1111/1755-0998.13022 (PMC6851627; doi:10.1111/1755-0998.13022)
Supplement: Supplementary file 6 [file MEN-19-900-s006.docx]

**Appendix S5 - DNA Metabarcoding laboratory workflow:**

DNA extraction

Preservative ethanol was removed and malaise bulk samples were dried in a 70 °C oven overnight to evaporate off the residual ethanol. Dried specimens were ground with a sterilized pestle to homogenize the tissue. DNA extraction for all samples was carried out in a 90:10 solution of insect lysis buffer (buffer ATL, Qiagen DNEasy Tissue Kit, Qiagen, Hilden, Germany) and Proteinase K, which was added in sufficient amounts enough to cover the ground specimen tissue. Lysis was performed overnight in a 56 °C oven. Samples were then allowed to cool to room temperature, 200 µL aliquots of the lysate were taken, and DNA was extracted from them using the DNEasy Blood & Tissue Kit (Qiagen, Hilden, Germany) following the manufacturer’s instructions.

Amplification of the CO1 barcode fragment

From each sample, 5 µL of extracted genomic DNA was used, along with Mango TAQ (Bioline, Luckenwalde, Germany), and High Throughput Sequencing (HTS) adapted mini-barcode primers (dgHco 5’-TAA ACT TCA GGG TGA CCA AAR AAY CA-3’, mlCOIintF 5’-GGW ACW GGW TGA ACW GTW TAY CCY CC-3’, Leray et al., 2013) were applied for PCR. Amplification success and fragment length were observed using gel electrophoresis. Amplified DNA was cleaned up (see https://zsm-entomology.de/wiki/The_Beetle_D_N_A_Lab ) and resuspended in 50 µL molecular water for each sample before proceeding. Illumina Nextera XT (Illumina Inc., San Diego, USA) indices were ligated to the samples in a second PCR reaction applying the same annealing temperature as for the first PCR reaction but with only 7 cycles, and ligation success confirmed by gel electrophoresis. DNA concentrations were measured using a Qubit fluorometer (Life Technologies, Carlsbad, USA), and samples were combined into 40 µL pools containing equimolar concentrations of 100 ng each. Pools were loaded into a 1% agarose gel, run at 90V for 45 minutes, bands of the target amplicon size of 520bp were excised with sterilized razor blades, and purified with a GeneJet Gel Extraction kit (Life Technologies, Carlsbad, USA), following the manufacturer’s instructions. A final elution volume of 20 µL was used. High-Throughput Sequencing (HTS) was performed on an Illumina MiSeq using v2 (2*250bp, 500 cycles, maximum of 20mio reads) chemistry.

Pre-processing and clustering of sequence data

All sample FASTQ files in this study were combined although they had been sequenced on separate runs throughout the study period. Two samples were unable to be identified by their file names, and were therefore excluded from the analysis. Sequence processing was performed with the VSEARCH v2.4.3 suite [(Rognes et al. 2016)](https://paperpile.com/c/to66Ry/Q1zb) and cutadapt v1.14 [(Martin 2011)](https://paperpile.com/c/to66Ry/e8hP). Due to not all of the runs yielding reverse reads of high enough quality to enable paired-end merging, only forward reads were utilized. Forward primers were removed with cutadapt. Quality filtering was with the fastq_filter program of VSEARCH, fastq_maxee 2, minimum length of 100 bp. Sequences were dereplicated with derep_fulllength, first at the sample level, and then concatenated into one fasta file, which was then dereplicated. Chimeric sequences were filtered out from the large fasta file using uchime_denovo. Remaining sequences were clustered into OTUs at 97% identity with cluster_size, and OTU table created with usearch_global. To reduce likely false positives, a cleaning step was employed which excluded read counts in the OTU table of less than 0.01% of the total numbers of reads of their respective samples [(see Elbrecht and Steinke 2018)](https://paperpile.com/c/to66Ry/dQli).
